# Exploring mental functions utilised by male youth team-based ball-sport athletes within academy programs: a systematic review and meta-aggregation

**DOI:** 10.3389/fspor.2024.1453817

**Published:** 2024-10-17

**Authors:** Joshua P. Whitty, Bon Gray, Nikki Milne

**Affiliations:** Faculty of Health Sciences and Medicine, Bond Institute of Health and Sport, Bond University, Robina, QLD, Australia

**Keywords:** youth, sports, psychological, development, mental functions

## Abstract

The rise of youth sport academies has led to a focus on long-term athletic development, and exploration into holistic approaches that incorporate psychological attributes to form biopsychosocial frameworks. The primary aim of this systematic review was to profile the psychological attributes of male youth team-based ball-sport athletes in academy-based programs and explore interactions between psychological attributes and athlete participation through the biopsychosocial model, the International Classification of Functioning, Disability and Health framework. This systematic review was conducted according to the Preferred Reporting Items for Systematic Reviews and Meta-analyses (PRISMA) guidelines. Six databases; PubMed, SPORTdiscus, Proquest, PsychINFO, Embase and Scopus were searched for relevant publications from root to 14th March 2024. The search returned 3306 records, and after applying the eligibility criteria 51 studies were included at full text and were critically appraised utilising the Mixed Methods Appraisal Tool. Data synthesis revealed 178 mental functions measured across 12 ICF categories, via 66 psychometric tools. Meta-aggregation revealed mean scores for 34 mental functions across eight ICF categories and 12 sub-categories. Male youth team-based ball-sport academy athletes display high levels of orientation to self and high energy levels; moderate to high levels of motivation and self-regulation; moderate levels of psychosocial functions, conscientiousness, regulation of emotion, and organising and planning. Low levels of clinical indicators and burnout were observed. The ICF framework can be utilised as a biopsychosocial framework for sport practitioners looking to profile the multidimensional and complex attributes of team-based ball-sport youth athletes in academy programs.

**Systematic Review Registration:** https://doi.org/10.17605/OSF.IO/9CE24.

## 1 Introduction

Significant financial investments are made by professional sporting clubs to identify and develop athletes within their respective talent academy programs (1–5). The primary aim of talent academy programs is to progress players from their respective academy lists to the club's top team (6, 7). As a result, research into athletic development in talent academies has intensified as professional teams look to fast-track athletes into their associated professional top tier squads (1, 2).

Talent academy programs provide an opportunity to systematically develop key attributes required for athletes to perform at the top level in their sport (8). The systematic process of development offers advantages for youth players to gain experience from high-level coaches (9) and performance staff and can lead to training exposure with professional squads, providing stimulus to fast-track player development (8). Conversely, the same talent development process may also have disadvantages such as intensified training and early specialisation that may, if not closely monitored and supported, lead to injury, burn-out, dropout, increasing perceived burden of selection and deselection pressures, leading to performance anxiety and other psycho-social issues (10–13). Aspiring youth players can be selected into talent programs from the age of 12 years or younger and progress through the talent pathway until approximately 19 years or older. Many critical points occur along the talent pathway where an athlete may be selected, deselected, or re-selected into talent programs, highlighting the importance for a long-term athletic development (LTAD) approach (4, 12, 14, 15).

Lloyd et al. (15) defines LTAD as “the habitual development of athleticism over time to improve health and fitness, enhance physical performance, reduce the relative risk of injury, and develop confidence and competence in youth”. To provide a LTAD framework for academies several talent and athletic development models currently exist, with a recent review finding 17 models across sporting and other contexts (16). Such models include; Differentiated Model of Giftedness and Talent (17), Developmental Model of Sports Participation (18), Balyi's Long Term Athlete Development (19), Youth Physical Development Model (20) and Foundations, Talent, Elite Mastery framework (21), with each considering development across a variety of biopsychosocial domains. These models provide significant insights and guidelines into the development of youth and offer practitioners working in the field, guidelines to facilitate individual talent development programs (i.e., sports academies) (14). The above-mentioned models show how multidimensional, dynamic, and complex the talent development pathway can be. The Composite Youth Development (CYD) model proposed by Lloyd et al. (14) highlights a blended approach between talent and athletic development models. The model integrates elements of age periods, maturation, talent, psychosocial, and physical development across the lifespan. A novel element of the CYD model, is the authors efforts to integrate psychosocial attributes into the framework The authors of the CYD model note that many important psychosocial attributes exist for each stage of development, and those selected in this model were based on available research and personal experiences (14). Further exploration of the literature is required to see what other psychological attributes maybe significant at the youth stage.

While physical attributes of talented athletes are widely documented (22–27), recent research has increasingly focused on the psychological attributes of athlete development in talent academies and the psychological domains that impact youth athletic development. Previous systematic reviews have described some of these psychological attributes utilised by talented youth athletes, which may help facilitate athlete development (1, 28–30). For example, Dohme et al. (30) found eight psychological skills [e.g., goal-setting, social support seeking, (pre) performance routine, self-talk] and eleven characteristics (e.g., self-confidence, hard-work ethic, resilience, focus) were facilitative for youth development. While Gledhill et al. (28) found 22 psychological factors related to talent development, with self-regulation, resilience, commitment and discipline emerging as the most influential on player development. The large number of psychological attributes that may be influential on the athletic development of these talented youth athletes warrants further investigation and profiling the psychological attributes as well as identifying the most reliable tools to assess the attributes is an important first step.

Furthermore, some studies have explored more complex interactions between psychological attributes and physical attributes (31–34), as well as, technical (35), and tactical abilities (36) of talented youth athletes. This provides evidence for the need of biopsychosocial frameworks to be utilised to view talented youth athletes in development pathways (1, 29, 37). By exploring the complex interactions and relationships between these attributes during development, a greater understanding of a holistic multidimensional approach can be achieved by sport practitioners working in the talent development field. Psychological attributes that may influence and lead to effective athletic development have long been considered, through the complex interactions between the athlete, task, and environment (28). Yet a cohesive, consistent, and practical framework for reporting these collective whole-of-person attributes in a sporting context is lacking which may reduce the utility of this information by sport practitioners, limiting holistic development of academy athletes.

**Figure 1:**
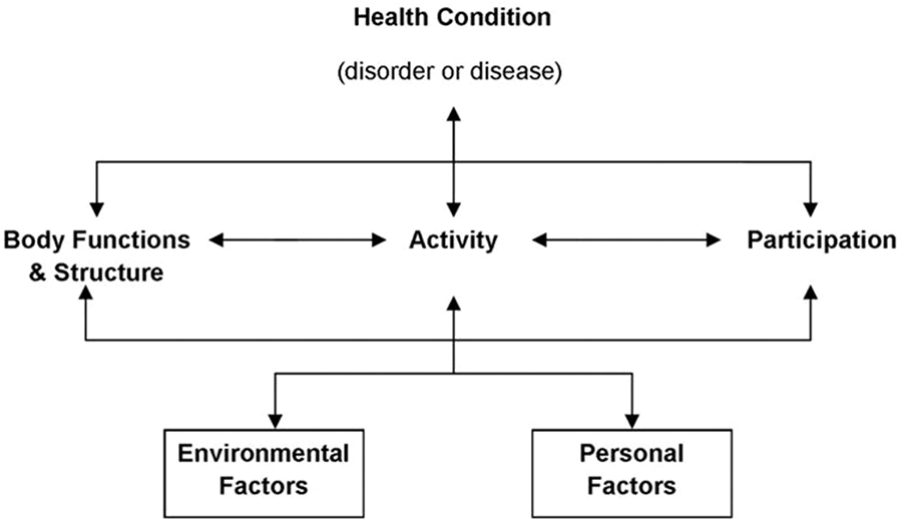
ICF framework (CC BY-NC-SA 3.0 IGO) (36).

A potential solution could be the use of the International Classification of Functioning, Disability and Health Framework (38). The ICF biopsychosocial framework provides common language and terminology for documenting the health and health-related states, outcomes, and determinants, as well as changes in health and developmental status, and functioning of individuals (38). Whilst commonly considered a framework for the health and disability sectors, the ICF framework conceptualises an individual across three domains of function: body structure and function (anatomical, physiological, and psychological), activity (skill ability and limitations) and participation (access to or restrictions in life activities at various levels, e.g., sport). Further the ICF contextualises an individual's level of functioning according to their environment and personal factors and identifies barriers and facilitators from these factors, which are likely to impact the three domains of function defined above (38). The ICF also has the utility to transition across an individual's lifespan, with consistent use of language, allowing greater emphasis on long-term development by all involved with athlete development as discussed by Elferink-Gemser et al. (39). Figure 1 provides a visual concept of the ICF as a biopsychosocial framework.

The ICF; an established biopsychosocial framework, could be used to view youth athletic development to explore the complex interactions (i.e., between biological, physiological, and psychological attributes and their sport-related activities and participation outcomes) that occur during the development of an individual athlete in the environment of team-based youth sport Academy programs whilst considering a LTAD approach (38).

The primary aims of this systematic review were to (i) profile the psychological attributes (referred to as mental functions at the body function level of the ICF) of male youth team-based ball-sport athletes in academy programs, and (ii) to explore interactions between psychological attributes (mental functions) of academy athletes and attributes in other ICF domains. A secondary aim was to profile the tools used to measure mental functions (i.e., psychological attributes), that sport practitioners working in the field may apply. The present systematic review was planned to extend the current knowledge regarding mental functions of male youth athletes participating in team-based ball-sport academy programs. A deeper understanding of mental function profiles of academy athletes and their potential influence in athletic talent development (for example, on participation outcomes) can guide and inform both researchers and health and sport practitioners working in the field of athlete development (28).

## 2 Method

This systematic review was conducted according to the Preferred Reporting Items for Systematic Reviews and Meta-analyses (PRISMA) guidelines (40). This systematic review was registered prior to data extraction with Open Science Framework (OSF) on the 9th February 2023 https://doi.org/10.17605/OSF.IO/9CE24.

### 2.1 Information and search strategy

The search strategy was developed in consultation with the primary authors and university librarian. Six databases: PubMed, SPORTdiscus, Proquest, PsychINFO, Embase and Scopus were searched for relevant publications. The search strategy included the use of synonyms and subject headings (e.g., MeSH terms), related to the four key concepts that have been aligned to the Population (P), Intervention (I), Comparison (C), and Outcome (O) framework; (P) male youth athletes, (I) talent academy programs, (C) team-based ball sports, (O) psychological attributes/mental functions (see Table 1).

**Table 1:** Search strategy.

Database	Search Strategy
Pubmed	(psycholog*[tiab] OR personality[tiab] OR “mental toughness”[tiab] OR grit[tiab] OR resilience[tiab] OR coping[tiab] OR “emotional intelligence”[tiab] OR “emotional competence”[tiab] OR self-regulation[tiab] OR self-control[tiab] OR imagery[tiab] OR motivation[tiab] OR “intrinsic motivation”[tiab] OR “fear of failure”[tiab] OR perfectionis*[tiab] OR passion[tiab] OR determination[tiab] OR commitment[tiab] OR self-confidence[tiab] OR self-efficacy[tiab] OR self-determination[tiab] OR “goal orientat*”[tiab] OR “Individuality”[Mesh] OR “Adaptation, Psychological”[Mesh] OR “Child Development”[Mesh] OR “Resilience, Psychological”[Mesh] OR “Emotional Intelligence”[Mesh] OR “Self-Control”[Mesh] OR “Imagery (Psychotherapy)”[Mesh] OR “Motivation”[Mesh] OR “Risk-Taking”[Mesh] OR “Perfectionism”[Mesh] OR “Acceptance and Commitment Therapy”[Mesh] OR “Self Concept”[Mesh] OR “Self Efficacy”[Mesh] OR “Personal Autonomy”[Mesh] OR “Goals”[Mesh] OR “Psychology, Sports”[Mesh]) AND (youth*[tiab] OR adolescen*[tiab] OR teen*[tiab] OR pubescent[tiab] OR young*[tiab] OR child*[tiab] OR juvenile*[tiab] OR “Adolescent”[Mesh]) AND (player*[tiab] OR athlete*[tiab] OR “Athletes”[Mesh]) AND (talent*[tiab] OR academy[tiab] OR gifted*[tiab] OR aptitude[tiab] OR “Aptitude”[Mesh] OR “Academies and Institutes”[Mesh]) AND (“team sport*[tiab]” OR game[tiab] OR football*[tiab] OR soccer[tiab] OR rugby[tiab] OR league[tiab] OR basketball[tiab] OR baseball[tiab] OR cricket[tiab] OR hockey[tiab] OR “Football”[Mesh] OR “Soccer”[Mesh] OR “Youth Sports”[Mesh] OR “Sports”[Mesh] OR “Basketball”[Mesh] OR “Baseball”[Mesh] OR “Hockey”[Mesh])

Key concepts including synonyms were added into Bond University's Systematic Review-Accelerator online polyglot search translator (http://sr-accelerator.com/) to translate the PubMed search string across remaining databases. Subject headings and title and abstract search functions were added to the search string, with the search for relevant literature initially conducted on the 24th of July 2022, with a secondary search undertaken on the 9th of February 2023 and a final search completed on the 14th of March 2024.

### 2.2 Data management, screening and study selection

**Figure 2:**
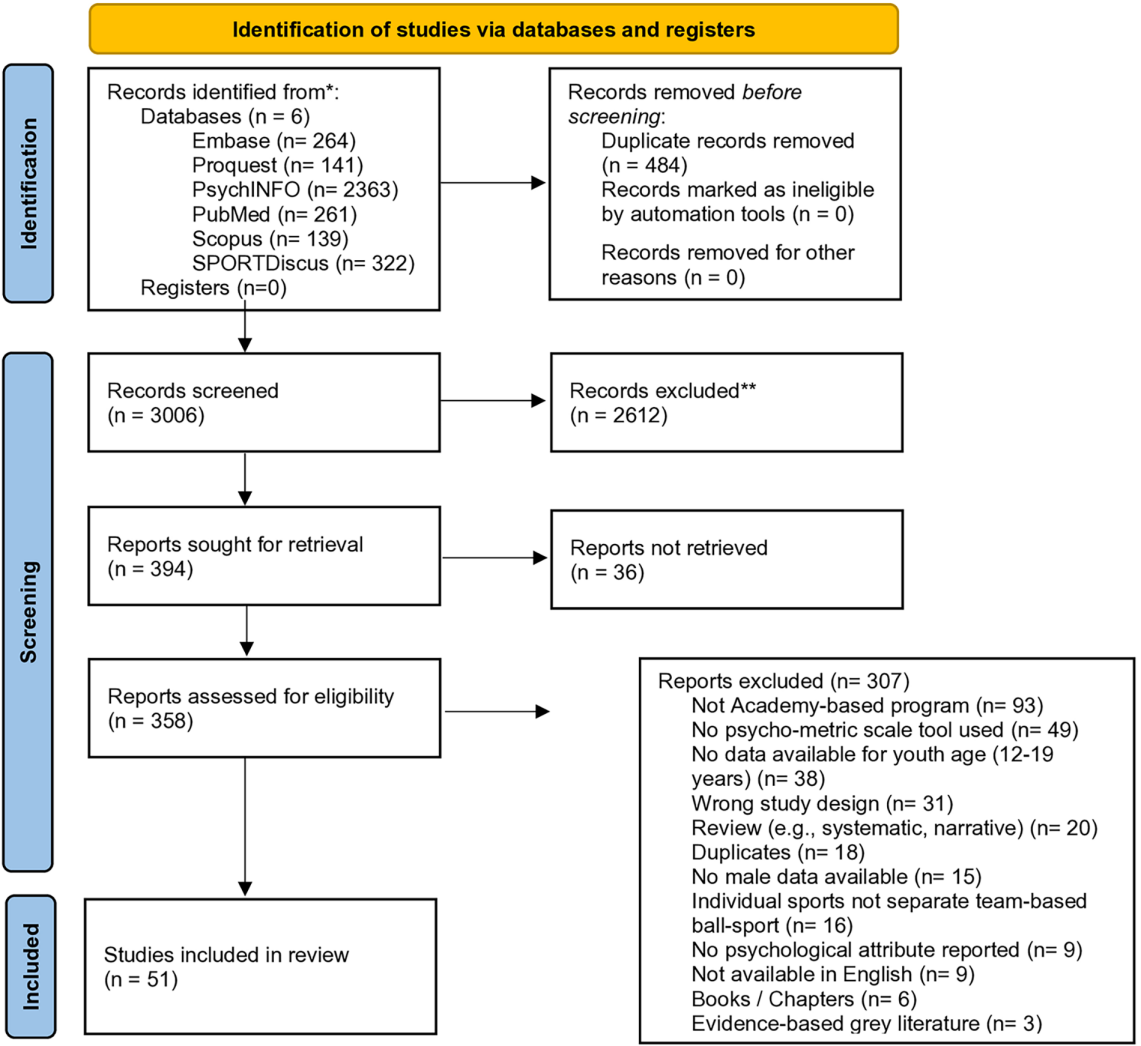
PRISMA flow diagram (38).

Results of the literature search were exported into the electronic management software program Endnote (version X8.0.2). Literature was then imported into the web-based software platform, Covidence (41) for screening and selection. Duplicates were automatically removed by the Covidence software. Two authors (JW and NM) independently performed title and abstract screening via the online Covidence platform. For studies that appeared to meet the inclusion criteria, or for studies where it remained unclear full text versions were retrieved. Full text articles were then screened against the eligibility criteria (Table 2) with any discrepancies resolved via discussions between the two reviewers until consensus was achieved. Results from the screening and selection process including reasons for exclusion at full text were recorded in accordance with PRISMA guidelines (40) and were documented via PRISMA Flow Diagram (Figure 2).

**Table 2:** Inclusion and exclusion criteria.

Inclusion Criteria	Exclusion Criteria
• Studies must include participants who were: - youth athletes, aged between 12 and 19 years of age (or population with mean age between these years) - male - involved in team-based ball sports (including stick/bat and ball/puck sports) - participate in academy-based development program • Studies must report at least one psychological attribute/mental function relating to talent development or sports performance • Studies must state validated psychometric tool used • Studies must be written in English • Peer reviewed studies (e.g., cross-sectional, observational, longitudinal)	• Study not available in full text • Individual sports or separate team-based sport data not available when multiple sports included. • Psychomotor attributes only • Athletes with a disability • Gaming athletes • Athletes with a documented neuro-developmental condition (e.g., ADHD, DCD, ASD) • No male data available for extraction in mixed sex studies • No data available for youth age (12-19 years) when mixed age study was included • Individual case studies • Data from retrospective interviews during adulthood based on youth experiences • Books/Chapters • Editorials/letter to editor/opinions/consensus statement • Review (e.g., systematic, narrative) • Evidence-based grey literature articles covering the above-mentioned inclusion criteria (e.g., policies, guidelines, procedures, articles, publicly available data)

### 2.3 Eligibility criteria

After establishing the key search terms and eligibility criteria using the PICO framework, studies were screened using the eligibility criteria outlined in Table 2.

### 2.4 Critical appraisal of methodological quality

The Mixed Methods Appraisal Tool (MMAT) (42) was used to assess the methodological quality of included studies, with each publication being reviewed independently by JW and NM. Kappa statistic was calculated between the two reviewers to determine the level of agreement in critical appraisal scores using SPSS software (43). Any discrepancies in critical appraisal scoring were discussed between appraisers to achieve consensus for final scores to determine the methodological quality of included articles.

The MMAT was used as it appraises the quality of: qualitative research, randomised control trials, non-randomised studies, quantitative descriptive studies and mixed-methods studies (42). The MMAT has been demonstrated to be a reliable tool and utilised successfully in previous research investigating psychological attributes within the sports literature (28). The scoring criteria was assessed (research questions, sample population, data quality, risk of bias and study adherence) using: “yes” scored as “1” and “no” or “can’t tell” scored as “0”.

### 2.5 Data extraction

Data extraction was completed by the primary author (JW) using a standardised data extraction table and managed electronically in a spreadsheet using Microsoft Excel (version 2016). This included: author/s, year of publication, title, aims, participant details (sample size, age range, sport, academy details and country of origin), psychometric tools used, type of psychometric value, psychometric value of the tool, source of psychometric value, mental function (psychological attribute) examined, descriptive statistics reported in the study, results relevant to study aims and MMAT scores. All data extracted was checked for accuracy by a second reviewer (NM). Where further descriptive data was required from the study the primary author emailed corresponding authors.

### 2.6 Data synthesis and analysis

A descriptive synthesis of extracted data was conducted to explore mental functions examined within talent academy programs from included studies. Only studies that attained a score of five or more out of seven on the MMAT were deemed to have robust methodology and were therefore included in the descriptive synthesis and meta-aggregation, and this method is consistent with previous methods applied in empirical research (44).

**Figure 3:**
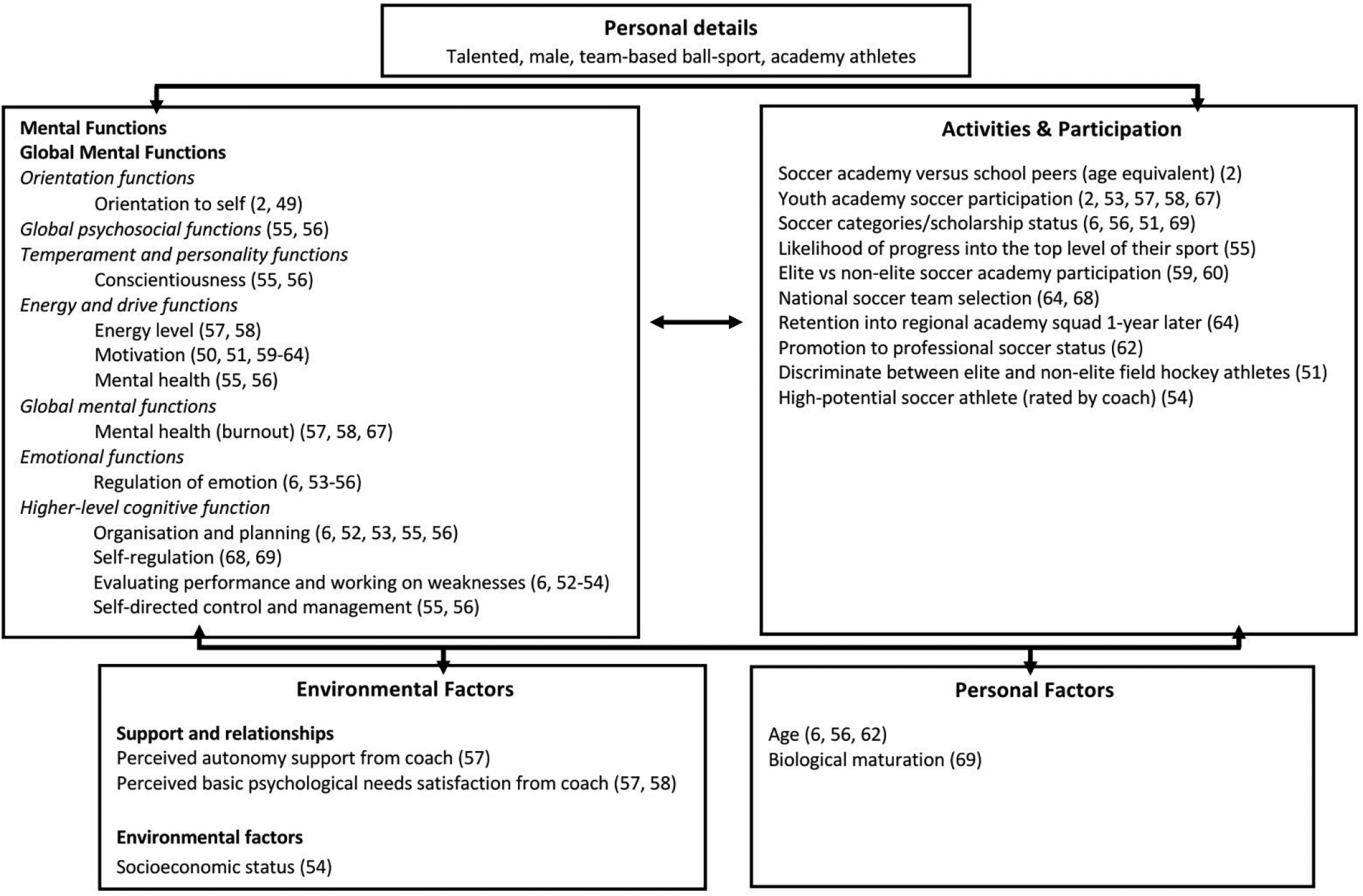
Interactions between athlete mental functions and participation in talented male team-based ball-sport athletes utilising the ICF fromework.

Mental functions (i.e., psychological attributes) were grouped according to ICF classifications and definition for each mental function (according to ICF language) for ease of interpretation. Where definitions were not available through ICF language, sports psychology literature was utilised. Descriptive synthesis involved the mental functions reported in individual studies being extracted and presented according to ICF classifications (45) with mental function tools also mapped according to the ICF framework. The grouping and mapping process according to ICF classifications in the descriptive synthesis was completed independently by one reviewer (JW) and validated by a second reviewer (NM). Interactions explored between the mental functions and other variables reported in the individual studies were documented and mapped according to the relevant ICF domains (Figure 3) and these interactions were narratively reported in the results section.

Attributes mapped to contextual “environmental and personal factors” related to physical, social and attitudinal environment where individuals live and conduct their lives (e.g., parents or coaches of athletes) (38). Attributes mapped to “personal factors” are specific background features of the individual (e.g., facilitators and barriers to participation in academy based Australian football programs) (38).

A meta-aggregation was conducted using data exacted on mental functions when more than one study used the same psychometric tool. An excel worksheet was utilised to calculate weighted means (using the weighted mean formula) and pooled deviation score (using the pooled standard deviation formula) for mental functions, (accounting for sample size in each study), when two or more studies reported mean scores for the same mental function. In studies that reported data at multiple timepoints, descriptive data from the first timepoint only was used, and repeated samples of the same population were not utilised in the meta-aggregation. This method was utilised to minimise missing data points and study withdrawals and to ensure a single sample was not over-represented leading to sampling bias. However, reported data that included different/new participants for multiple age groups were included. For studies that included sub samples of participants (i.e., different ages, selected/deselected academy participants or different scholarship status) an intra-study weighted mean and standard deviation was calculated to provide the descriptive statistics for the individual samples in that study.

Meta-aggregated data for psychological attributes was also expressed using ICF coding (38). Table 3 outlines how the meta-aggregated data based on different psychometric (Likert) scales, aligns to the language utilised in the ICF coding system (38). Language utilised in the psychometric tools measuring mental functions was themed to align with the ICF severity coding language of body function (specifically mental function). The intent of aligning the psychometric profiling scales to the ICF coding system, is to enable greater understanding of the relationships and interactions between various attributes assessed. By providing a standardised representation of the meta-aggregated results this can enhance meaningful interpretations between multiple attributes and domains. For example, if the meta-aggregated mean on a 1-to-5 scale psychometric tool is 1, it will be mapped into the ICF as “none”, “absent” or “negligible”.

**Table 3:** Alignment of data from psychometric measures to ICF coding system.

ICF descriptor/qualifier (38)	ICF descriptor code (38)	Scales from Psychometric Tools			
		0 to 4 scale	1 to 5 scale	1 to 6 scale	1 to 7 scale
None, absent, negligible	0 (0–4%)	0	1	1	1
Mild, slight, low	1 (5–24%)	1	2	2	2
Moderate, medium, fair	2 (25–49%)	2	3	3–4	3–4
Severely, high	3 (50–95%)	3	4	5	5–6
Total, complete	4 (96–100%)	4	5	6	7

## 3 Results

### 3.1 Study selection

The PRISMA flow diagram (Figure 2) provides a summary of the search outcomes. The search of six databases resulted in 3,490 studies. After duplicate removal (*n* = 484), title and abstract screening was completed on 3,006 studies. Following full text screening against the inclusion and exclusion criteria 51 studies were included at full text for data extraction and were critically appraised (Figure 2 PRISMA statement). Supplementary Table S1 provides a full list of excluded studies after full text screening.

### 3.2 Methodological quality

The percentage agreement between the two reviewers during critical appraisal was high with 89.7%. Cohen's Kappa analysis demonstrated a skewed critical appraisal outcome to a “yes” response, representing a phenomenon known as “paradoxes of Kappa” indicating the Kappa statistic does not appropriately identify interrater reliability between assessors with this relatively small number of studies (*κ* = 0.364; *p* = 0.001) (46). However, based on the percentage agreement a high level of agreement in methodological quality between the independent reviewers can be concluded. Fifty of the 51 studies were considered high quality, based on achieving a score equal to or greater than 5/7 (71%) on the MMAT (44) and these 50 studies were utilised in the descriptive synthesis and meta-aggregation. Supplementary Table S2 provides the critical appraisal consensus scores for each study.

### 3.3 Study characteristics

Data extracted from all 51 studies are reported in Supplementary Table S3. From the 50 high quality studies a total of 6,998 participants were included in the present review. Soccer was the most investigated sporting academy (*n* = 42), followed by field hockey (*n* = 3) and cricket (*n* = 2). Rugby league (*n* = 1) and rugby union (*n* = 1) were also represented with one mixed study that included soccer, rugby league and rugby union participants. Male youth team-based ball-sport academy athletes from the United Kingdom (*n* = 23) and other European countries (*n* = 23) represented 92% of the studies included in this review. Australia (*n* = 4) was the other country represented.

One hundred and seventy-eight mental functions (psychological attributes) were examined across the 50 studies and were mapped to 12 ICF categories. These 12 ICF mental function categories include consciousness, orientation, global psychosocial, dispositions and intra-personal, temperament and personality, energy and drive, global mental function, others specified and unspecified, attention, emotion, thought, basic cognitive, higher-level cognitive. Of the 12 ICF categories, 31 sub-categories are represented. Supplementary Table S4 presents the mental functions reported from each study grouped to the ICF category and sub-category as well as the tools used to capture each mental function.

Sixty-six tools were used to assess the 178 mental functions. Twenty-four tools were used to assess energy and drive functions, and its sub-categories. Temperament and personality functions were measured through 16 psychometric tools. Emotional functions were quantified by 13 psychometric tools. Global mental functions, higher-level cognitive functions, global psychosocial functions, and orientation functions were assessed by nine, eight, six and four tools respectively. Four tools were utilised to measure attention and thought functions, while three tools were used to measure dispositions and intra-personal functions. Finally, one tool was identified to measure consciousness and basic cognitive functions.

The coefficient alpha or “Cronbach's alpha” was most utilised to determine the psychometric value of the tools (47) with 35 studies using this approach. Authors who reported the psychometric value of their psychometric tools reported an acceptable (48) level of reliability as *α* = >0.70. Ten studies did not report the psychometric value of the tool used or it remained unclear within their publication. Two studies used an Omega value, another used a test-retest reliability measure as the psychometric value, while 2 studies remained unclear.

Authors calculated their own psychometric value on 33 studies, seven studies used previously reported data and ten did not report any psychometric value.

### 3.4 Profile of mental functions (from psychometric measures)

Measures of mental functions extracted from 50 individual studies with high methodological quality are outlined in the data extraction Table (Supplementary Table S3) and each mental function has been mapped to relevant ICF categories and sub-categories (Supplementary Table S4). Meta-aggregated weighted mean results of mental functions for male youth team-based ball-sport academy athletes are documented in Tables 4–11.

**Table 4:** Meta-aggregated data for mental function, orientation function—orientation to self and others.

ICF category	ICF sub-category	Psychological attribute	Participants (Studies)	Mean	Standard deviation	ICF Descriptor	Practical interpretation based on aggregated mean results
Orientation functions	Orientation to self	Total athletic identity	201 participants (2, 49)	40.46/49	2.26	High	Male youth athletes in team-based academies highly identify themselves as athletes, which can lead to positive and negative psychosocial impacts.
		Total athletic identity—Exclusivity	201 participants (2, 49)	5.77/7	0.00	High	An individual’s self-worth is strongly established through sport participation for male youth athletes in team-based academies.
		Total athletic identity—Negative affectivity	201 participants (2, 49)	6.11/7	0.24	High	Youth athletes experience higher negative emotional responses as a result from unwanted sporting outcomes in youth team-based sports academies.
	Orientation to others	Total athletic identity—Social identity	201 participants (2, 49)	5.51/7	0.12	High	Youth athletes highly view themselves as holding the role of an athlete, when participating in team-based sports academies.
		Team emphasis	134 participants (50, 51)	3.15/5	0.85	Moderate	Male youth athletes in team-based academies focus on their individual performance as much as they do on team elements.

**Table 5:** Meta-aggregated data for mental function, global psychosocial functions.

ICF category	ICF sub-category	Psychological attribute	Participants (Studies)	Mean	Standard deviation	ICF Descriptor	Practical interpretation based on aggregated mean results
Global psychosocial functions	N/A	Support for long-term success	239 participants (6, 52–54)	4.41/6	0.34	Moderate	Male youth athletes in team-based academies perceive that they are being equipped with the necessary skills required to achieve long-term success.
		Seeking and using social support	566 participants (55, 56)	4.31/6	0.94	Moderate	Youth athletes adequately use their support networks to help facilitate their development in team-based sports academies.

**Table 6:** Meta-aggregated data for mental function, temperament, and personality functions (conscientiousness and confidence).

ICF category	ICF sub-category	Psychological attribute	Participants (Studies)	Mean	Standard deviation	ICF Descriptor	Practical interpretation based on aggregated mean results
Temperament and personality functions	Conscientiousness	Ability to organise and engage in quality practice	239 participants (6, 52–54)	4.93/6	0.10	High	Male youth athletes in team-based academies display high levels of attitudes and behaviours during practice that help facilitate effective development.
		Perfectionistic tendencies	566 participants (55, 56)	3.37/6	0.93	Moderate	Male youth athletes in team-based academies display modest levels of perfectionism, which may have both positive and negative traits.
	Confidence	Confidence	134 participants (50, 51)	3.56/5	0.84	High	Male youth athletes in team-based academies display high levels of self-confidence.

**Table 7:** Meta-aggregated data for mental function, energy, and drive functions.

ICF category	ICF sub-category	Psychological attribute	Participants (Studies)	Mean	Standard deviation	ICF Descriptor	Practical interpretation based on aggregated mean results
Energy and drive functions	Energy level	Subjective Vitality	195 participants (57, 58)	4.97/7	0.74	High	Male youth athletes in team-based sports academies reported elevated levels of being energetic and “feeling alive” from their participation in their sport academy.
	Motivation	Task orientation	568 participants (50, 59–62)	4.20/5	0.78	High	Male youth athletes reported being highly motivated by personal improvement, learning and effort within team-based sports academies.
		Ego orientation	568 participants (50, 59–62)	3.31/5	0.90	Moderate	Male youth athletes reported being moderately motivated by winning, external validation and comparing oneself to others within team-based sports academies.
		Win orientation	365 participants (62–64)	4.36/5	0.76	High	Male youth athletes in team-based sports academies reported a high desire to win, by focusing on the outcome, potentially at the expense of development.
		Goal orientation	385 participants (62–65)	4.55/5	0.68	High	Male youth athletes in team-based sports academies reported very high usage of setting specific and measurable objectives, working towards these goals and placing the importance on personal progress and improvement, regardless of the outcome.
		Motivation	134 participants (50, 51)	3.93/5	1.00	High	Male youth athletes participating in team-based academies reported high levels of motivation and a drive for excellence and success.
		Competitiveness	271 participants (62, 63)	4.51/5	0.69	High	Male youth athletes in team-based academies display a strong competitive nature, who possess a high desire to excel against others and be the best in their sport. As well as showing an appreciation for the challenges and opportunities presented in sport.
		Hope for success	377 participants (62, 66)	2.34/3	0.69	High	Male youth athletes in team-based academies show high levels of hope for success, indicating a positive outlook on their ability to achieve sporting success and challenging goals.
		Fear of failure	377 participants (62, 66)	0.62/3	0.71	Low	Male youth athletes in team-based academies scoring low on fear of failure, suggests an adaptive approach to training and competition. With athletes displaying higher levels of persistence, risk-taking and overall enjoyment in sports.
		Self determination (Index score)	114 participants (64, 65)	9.48/-18–18*	1.60	High	Male youth athletes in team-based academies reported high levels of self-determination, where athletes may be more intrinsically motivated, and autonomous, which can lead to greater persistence, goal achievement and overall satisfaction.
	Energy and drive functions, other specified, mental health	Clinical indicators	566 participants (55, 56)	2.34/6	0.93	Low	Male youth athletes in team-based academies reported low levels of mental health issues regarding anxiety, depression, eating disorders.

**Table 8:** Meta-aggregated data for mental function, global mental function, others specified and unspecified—mental health.

ICF category	ICF sub-category	Psychological attribute	Participants (Studies)	Mean	Standard deviation	ICF Descriptor	Practical interpretation based on aggregated mean results
Global mental functions, others specified and unspecified	Global mental functions, others specified, burnout	Exhaustion	264 participants (57, 67)	2.21/5	0.85	Low	Male youth athletes in team-based academies reported low levels of exhaustion in team-based sports academies may experience enhanced athletic performance, increased enjoyment, and satisfaction in training as well as overall well-being.
		Burnout	277 participants (58, 67)	2.10/5	0.72	Low	Male athletes reported low levels of burnout in team-based sports academies which can lead to improved athletic performance and well-being. Low levels of burnout can also enable the maintenance of a healthy balance between sports participation and other aspects of the athlete’s life.

**Table 9:** Meta-aggregated data for mental function, attention functions.

ICF category	ICF sub-category	Psychological attribute	Participants (Studies)	Mean	Standard deviation	ICF Descriptor	Practical interpretation based on aggregated mean results
Attention functions	Sustaining attention	Concentration	134 participants (50, 51)	3.43/5	0.90	Moderate	Male youth athletes in team-based academies demonstrate moderate levels of concentration, this could benefit on-field decision making and overall performance due to the ability to maintain focus, and process information effectively.

**Table 10:** Meta-aggregated data for mental function, emotional functions.

ICF category	ICF sub-category	Psychological attribute	Participants (Studies)	Mean	Standard deviation	ICF Descriptor	Practical interpretation based on aggregated mean results
Emotional functions	Regulation of emotion	Anxiety Control	134 participants (50, 51)	3.74/5	0.97	High	Male youth athletes in team-based academies reported a high tendency to control their anxiety levels. Which may enhance resilience, performance under pressure and improved emotional well-being.
		Coping with performance and developmental pressures	239 participants (6, 52–54)	4.07/6	0.79	Moderate	Male youth athletes in team-based academies reported moderate levels of coping with performance and developmental pressures. This could benefit athletes by increasing resilience, and overall well-being, enabling them to navigate the challenges and setbacks experienced during an academy pathway.
		Adverse response to failure	566 participants (55, 56)	3.01/6	0.95	Moderate	Youth athletes in team-based sports academies demonstrated a moderate level of adverse response to failure, which may lead to improved resilience, and greater learnings from setbacks.
		Active coping	566 participants (55, 56)	4.48/6	0.90	Moderate	Male youth athletes in team-based academies reported moderate levels of active coping, that could improve mechanisms such as self-regulation to navigate the challenges along the talent pathway.

**Table 11:** Meta-aggregated data for mental function, higher-level cognitive functions.

ICF category	ICF sub-category	Psychological attribute	Participants (Studies)	Mean	Standard deviation	ICF Descriptor	Practical interpretation based on aggregated mean results
Higher-level cognitive functions	Organisation and planning	Mental Preparation	134 participants (50, 51)	2.72/5	0.94	Moderate	Male youth athletes in team-based academies reported moderate levels of mental preparation, this can lead to effective use of mental skills and strategies such as mental rehearsal to handle competitive stress and optimise performance.
		Imagery use during practice and competition	239 participants (6, 52–54)	4.34/6	0.85	Moderate	Male youth athletes in team-based academies reported moderate levels of imagery use during practice and competition. This could enhance learning and skill acquisition, enable consistent performance through such imagery strategies.
		Imagery and active preparation	566 participants (55, 56)	3.95/6	0.94	Moderate	Male youth athletes in team-based academies reported moderate levels of imagery and active preparation for performance and arousal control purposes. This can improve mental readiness, skill execution, consistent performance.
	Higher-level cognitive functions, other specified, self-regulation	Self-regulation (global)	642 participants (68, 69)	3.92/5	0.80	High	Male youth athletes in team-based academies showed high levels of self-regulation that could lead to greater self-control, and emotional management. This allows the athlete to help regulate their thoughts, emotions and behaviours.
		Evaluating performance and working on weakness	239 participants (6, 52–54)	5.17/6	0.76	High	Male youth athletes in team-based academies demonstrated high levels of evaluating performance and working on weakness. This can create greater self-awareness, targeted skill development, as the athlete seeks feedback to identify areas of improvement and focus effort to improve that skill leading to continued progress.
		Self-directed control and management	566 participants (55, 56)	4.42/6	0.91	Moderate	Male youth athletes in team-based academies displayed moderate levels of self-directed control and management. This can improve personal accountability, decision making, as the athlete takes ownership of their actions, and actively contribute to their own development.

### 3.5 Interactions between psychometric variables according to ICF mental functions, and other domains of the ICF in male youth team-based ball sport academy athletes

This section narratively synthesises findings from studies that report on relationships between mental functions of male youth team-based ball-sport academy athletes and other domains of the ICF.

#### 3.5.1 Orientation function—orientation to self and others

Table 4 provides a meta-aggregated summary of orientation functions. Total athletic identity (Athletic Identity Measurement Scale—AIMS) did not differentiate level of academy participation (i.e., playing) in soccer with no significant differences noted in AIMS scores between players from different leagues (*F* = 0.5, *p* = 0.68) (49). Furthermore, the total athletic identity subscales (exclusivity, negative affectivity, social identity) do not differ between academy players from different clubs or leagues (49). Living arrangements did not have any significant effect on total athletic identity (*p* = 0.25) or associated sub-scales, nor did year of apprenticeship (*p* = 0.16) (49). However, soccer academy players have significantly higher total athletic identity (*p* < 0.01) compared to age-equivalent school peers and this is characterised by academy soccer players having consistently higher “exclusivity” scores (*p* ≤ 0.01) with social identity and negative affectivity not contributing to the differences (2). A players’ orientation towards “team emphasis” does not significantly discriminate between selected and non-selected academy soccer players (*p* = >0.05) (50), or between elite and sub-elite male youth field hockey players (51).

#### 3.5.2 Global psychosocial functions

Table 5 provides a meta-aggregated summary of global psychosocial functions. A player's belief in having “support for long-term success” [Psychological Characteristics for Developing Excellence Questionnaire (PCDEQ) Version 1 Factor 1] do not significantly differentiate talented academy soccer players who play up an age-group from those who do not (*p* = 0.32) (52). Similarly, Hauw et al. (53) identified that the PCDEQ (Version 1) factor “long-term performance support” could not be used to discriminate between good and poor developers in Swiss soccer academy players as the “long-term performance support” factor was not scored at a level expected for good developers (3.93/6 vs. 4.25/6), despite the participants being from two professional soccer academies. Saward et al. (6) found no significant effect of age or eventual scholarship status on a soccer players PCDEQ (Version 1) Factor 1 scores “support for long-term success”. Kelly et al. (54) found no significant difference between scores of higher or lower potential athletes “support for long term success” and socioeconomic status in English soccer academy athletes (*p* = 0.95). Finally, Hill et al. (55) identified that UK academy-based players whose coaches believed had a higher likelihood of progressing to the top level in their sport, had significantly higher “seeking and using social support” behaviour (PCDEQ—Version 2 Factor 5) than those whose coaches believed had a low likelihood of progressing to the top level (*p* = 0.005). Meanwhile, Barraclough et al. (56) reported that “seeking and using social support” behaviours were non-significant across age groups and scholarship categories, with only a medium effect size noted (*p* = <0.001) for older players within the category one academy group.

#### 3.5.3 Temperament and personality functions (conscientiousness and confidence)

Table 6 provides a meta-aggregated summary of temperament and personality functions. When exploring the attribute of conscientiousness, Saward et al. (6) demonstrated that there is no significant effect of age (*p* = >0.05) or eventual scholarship status (*p* = >0.05) on players “ability to organise and engage in quality practice” (PCDEQ—Version 1 Factor 4). Additionally, Kelly et al. (52) found that a players “ability to organise and engage in quality practice” (PCDEQ Version 1—Factor 4) is not significantly different between academy soccer players who played up an age level compared to those who did not (*p* = 0.50). Kelly et al. (54) found no significant differences between “higher” or “lower” potential athletes who had the ‘ability to organise and engage in quality practice’ and socioeconomic status in English soccer academy athletes (*p* = 0.07). When exploring perfectionistic tendencies, Hill et al. (55) demonstrated that UK academy-based players whose coaches believed they had a higher likelihood of progressing to the top level in their sport, had significantly higher ‘perfectionistic tendencies’ (PCDEQ—Version 2 Factor 4) than those whose coaches believed had a low likelihood of progressing to the top level (*p* = <0.01). ‘Perfectionistic tendencies’ significantly increased through the age groups (*p* = <0.001) and categories of participation (*p* = <0.001) with those in the most elite category demonstrating highest levels of perfectionism (56). Huijgen et al. (50) found no significant difference in “self-confidence” for adolescent soccer players in the Netherlands who were selected into talent development programs of professional soccer clubs, compared to those who were deselected (*p* = >0.05). Elferink-Gemser et al. (51) also found no significant difference in confidence between elite and sub-elite male youth field hockey players. However, Hauw et al. (53) found a significant difference in self-confidence levels (*p* = 0.16) between Swiss soccer players who were grouped (group 1) by scoring low on all traits except agreeableness and the who were grouped (group 3) by scoring medium on emotional stability and high on other traits.

#### 3.5.4 Energy and drive functions

Table 7 provides a meta-aggregated summary of energy and drive functions. Adie et al. (57) found that levels of “subjective vitality” increased (*p* = <0.05) across two youth soccer academy seasons when players perceived that they received autonomy support from their coach. Differences were observed at both an intrapersonal level (*p* = <0.001) and interpersonal level (*p* = <0.001). Similarly, Cheval et al. (58) noted that competence (*p* = <0.001), and autonomy (*p* = <0.001) positively predicted “subjective vitality” over a 3-month period for elite youth French soccer players.

Kavussanu et al. (59) reported significant differences in “task orientation” in elite vs. non-elite soccer academy players (*p* = 0.03). This was supported by findings from Reilly et al. (60) who found a significant difference between elite and sub-elite English soccer players in “task orientation” (*p* = <0.05). Elite status was described in both studies as players who were signed with a professional soccer club, whereas non-/sub-elite players were described as those who played at local or school level, that had not signed with a professional soccer club. However, when comparing players within an academy program, Bennett et al. (61) found no significant difference between tier 1 and tier 2 academy players in “task orientation” for both early adolescent (*p* = >0.05) and mid-adolescent (*p* = >0.05) Australian youth soccer players. This was also supported by Huijgen et al. (50) who observed no significant differences in “task orientation” for soccer players who were selected into talent development programs of professional soccer clubs in the Netherlands, compared to those who were deselected (*p* = >0.05). Finally, Wachsmuth et al. (62) found no significant difference in task orientation between under-12 and under-17 male German soccer academy athletes, as well as no prognostic value on under-17 players achieving professional soccer status.

Wachsmuth et al. (62) found significantly higher scores in ego orientation for under-17 male youth soccer athletes when compared to under-12 athletes (*p* = < 0.001). However, ego orientation did not differentiate between elite vs. sub-elite players (*p* = ns) (59), and (*p* = > 0.05) (60). Nor did it differentiate between tier 1 or tier 2 academy players (*p* = > 0.05) (61), or selected or deselected soccer players (*p* = >0.05) (50). Ego orientation did not predict the likelihood of under-17 male soccer athletes progressing into professional status (*p* = > 0.05) (62).

Joseph et al. (63) found no significant difference in win orientation scores between selected and non-selected under-16 (*p* = 0.58) and under-18 (*p* = 0.16) players in the basketball talent pathway program. However, Zuber et al. (64) found that win oriented failure fearing youth soccer players were 1.8 times more likely to remain in the regional squads a year later, but would not be selected into the National under-15s squad. Wachsmuth et al. (62) also found, win orientation scores were significantly higher in under-17 male youth soccer athletes when compared to under-12 athletes (*t* = 13.69, *p* = < 0.001). However, win orientation did not significantly influence a youth athlete's progression into professional status (62).

No significant differences were found in goal orientation scores between selected and non-selected under-16 (*p* = 0.55) and under-18 (*p* = 0.99) basketball talent pathway athletes (63). Sieghartsleitner et al. (65) also found psychological characteristics of which goal orientation was part, did not significantly influence the holistic model (*p* = 0.80) or the multidimensional model (*p* = 0.27) that predicted talent selection in youth soccer. Wachsmuth et al. (62) demonstrated no significant difference between age groups (under-12 and under-17) in a youth German soccer academy, nor did goal orientation scores distinguish between under-17 soccer athletes and their progression into professional status. In contrast Zuber et al. (64) explained that when players were compared to average motivated players the intrinsic achievement-oriented players were 1.7 times more likely to remain in the program the following year. These players had 1.7 times higher odds of being selected in the under-15 national team than the average motivated player.

Wachsmuth et al. (62) found under-17 male German soccer academy athletes who scored higher in competitiveness were 3.93 times (*p* = < 0.05) more likely to be promoted to professional status as a soccer player than those who scored lower. However, Joseph et al. (63) found no significant difference between levels of competitiveness scores in selected vs. non-selected under-16s (*p* = 0.90) and under-18s (*p* = 0.54) in male Australian basketball youth athletes.

Under-17 German male soccer academy athletes who scored higher in hope for success were found to be 3.25 times more likely to progress into professional status (*p* = < 0.05) (62). Further, hope for success was seen to provide a significant predictor of an athletes performance as rated by their coaches in Swiss male youth soccer athletes (*p* = 0.03) (66).

Under-17 German male youth soccer academy athletes with low levels in fear of failure were 4.57 times more likely to progress to professional status (*p* = < 0.05), than those who scored highly. Zuber and Conzelmann (66) found fear of failure scores did not significantly impact on performance as rated by their coaches in a male Swiss academy cohort.

“Motivation” was identified by Elferink-Gemser et al. (51) as one of four variables that would successfully discriminate between talented elite and sub-elite field hockey players [accounting for 46% of the variance in group membership (i.e., elite participation group)]. However, Huijgen et al. (50) found no significant differences in motivation levels between selected and deselected players in a soccer talent development program (*p* = > 0.05).

Sieghartsleitner et al. (65) demonstrated mental functions that included self-determination, did not significantly influence the holistic model (*p* = 0.80) or the multidimensional model (*p* = 0.27) that predicted talent selection in youth soccer. Whereas, Zuber et al. (64) reported Swiss soccer players who scored highest in self-determination were those players clustered in the high intrinsic achievement-oriented players who were more likely to remain in the squad and more likely to be selected in the national team.

Hill et al. (55) showed UK academy-based players who were rated by their coaches as having a higher likelihood of progressing to the top level in their sport, had significantly lower “clinical indictor” (PCDEQ—Version 2 Factor 7) scores than those whose coaches believed had a low likelihood of progressing to the top level (*p* = <0.01). Barraclough et al. (56) found significant differences between clinical indictor scores across different levels of participation (category 1, 2, 3 and grass roots) (*p* = 0.001) for youth soccer academy participants. However, no significant differences were seen between age groups (*p* = 0.52).

#### 3.5.5 Global mental function, others specified and unspecified—mental health

Table 8 provides a meta-aggregated summary of global mental functions. Adie et al. (57) found that levels of exhaustion were significantly different between elite youth soccer academy players, when players reported lower levels of autonomy from their coaches, and this was linked to higher emotional and physical exhaustion (*p* = < 0.001). Exhaustion levels were seen to increase over the course of season one for elite youth soccer academy players (*p* = < 0.01) (57). No significant relationships appeared at the within person level between basic psychological needs and emotional and physical exhaustion (57). However, a significant interaction between relatedness to others and how exhaustion changes over time, helped predict emotional and physical exhaustion levels (*p* = < 0.01) (57). Curran et al. (67) found when professional youth soccer players from the UK, had their psychological needs meet, they were less likely to feel emotionally and physically exhausted (*p* < 0.01).

Cheval et al. (58) found that for elite French soccer players, coach autonomy support changed over time and when players felt less supported by their coach in making decisions, the likelihood of players experiencing burnout increased (*p* = 0.022). Cheval et al. (58) also found the levels of soccer players satisfaction or frustration for the need of competence (*p* = 0.001), and autonomy (*p* < 0.001) led players to experience a higher level of burnout. Likewise, when players perceived they had too much control they were also likely to experience burnout (*p* = 0.005) (58). Similarly, Curran et al. (67) found that professional youth soccer players in the UK were less likely to experience burnout when their psychological needs were meet (*p* < 0.01). Curran et al. (67) also found a small but significant negative correlation between harmonious passion and burnout (*r* = −0.17, *p* = < 0.05) in professional youth soccer players in the UK.

#### 3.5.6 Attention functions

Table 9 provides an aggregated summary of attention function. Concentration scores did not discriminate between elite (3.46 ± 0.39) and sub-elite (3.46 ± 0.71) talented male youth field hockey players (51). Likewise, Huijgen et al. (50) found no statistically significant difference in concentration between talented soccer players who were selected or deselected from a talent development program in the Netherlands (*p* > 0.05).

#### 3.5.7 Emotional functions

Table 10 provides an aggregated summary of emotional functions. Anxiety control scores did not differentiate between elite (4.04 ± 0.48) and sub-elite (3.94 ± 0.64) talented male youth field hockey players (51). Huijgen et al. (50) also found no statistically significant differences in the ability to control anxiety for talented soccer players who were selected or deselected from a talent development program in the Netherlands (*p* = > 0.05).

Saward et al. (6) demonstrated that scores for “coping with performance and developmental pressures” (PCDEQ—Version 1 Factor 3) significantly increased with age (*p* = < 0.05) within male youth soccer academy players. Players who would go on to receive a category 1 or 2 scholarship also scored significantly higher in coping with performance and developmental pressures than those who would receive a category 3 or 4 scholarship, or no scholarship (*p* = < 0.05). Kelly et al. (54) found significantly higher-potential athletes (as rated by their coach) had scored greater for “coping with performance and developmental pressures” when the athletes were from lower socioeconomic status than those of higher socioeconomic status (*p* = 0.01). However, Hauw et al. (53) noted that scores could not be used to discriminate between good and poor developers in Swiss soccer academy players as the factor was not scored at a level expected for good developers (4.02/6 vs. 4.27/6), despite the participants being from two professional soccer academies. Additionally, Kelly et al. (52) found that a player's score in the ability of “coping with performance and developmental pressures” (PCDEQ Version 1—Factor 3) did not significantly differ between academy soccer players who played up an age level compared to those who did not (*p* = 0.11).

Hill et al. (55) demonstrated that UK academy-based players across soccer, rugby league and rugby union, whose coaches believed had a higher likelihood of progressing to the top level in their sport, had significantly higher “adverse response to failure” behaviours (PCDEQ—Version 2 Factor 1) than those players whose coaches believed had a low likelihood of progressing to the top level (*p* = < 0.001). Barraclough et al. (56) found small significant effects between age groups on adverse response to failure (*d* = 0.49, *p* = < 0.001), with older players in under 16s and youth soccer teams scoring highest. A medium effect size was seen between levels of participation (*d* = 0.59, *p* = < 0.001), where category 1 soccer academy players scored higher in adverse response to failure (56).

Barraclough et al. (56) found small significant differences in “active coping” behaviour (PCDEQ—version 2 Factor 6) between top tier academy categories (*p* = < 0.001), however no significant differences were observed between age groups (*p* = 0.82). Hill et al. (55) observed significantly higher mean scores for “active coping” behaviours (PCDEQ—Version 2 Factor 1) in the group of academy players (soccer, rugby league and rugby union) who coaches believed had a higher likelihood of progressing to the top level in their sport, when compared to players whose coaches believed had a low likelihood of progressing to the top level (*p* = < 0.001).

#### 3.5.8 Higher-level cognitive functions

Table 11 provides an aggregated summary of high-level cognitive functions. Elferink-Gemser et al. (51) reported no difference in the mental preparation scores between elite (2.22 ± 0.64) verse sub-elite (2.31 ± 0.75) talented male youth field hockey players. No statistically significant differences were observed in the use of mental preparation between soccer players who were selected or deselected from a Dutch development program (*p* > 0.05) (50).

The use of imagery during practice and competition (PCDE—version 1, factor 2) was found to significantly decrease with age (*p* = < 0.05) for category 2 English soccer academy players (6). However, Kelly et al. (52) found no significant difference between English academy soccer players who played up an age, compared to those who didn’t within the tier 4 academy (*p* = 0.84). Similarly, Kelly et al. (54) found no significant differences between “higher” or “lower” potential athletes who scored higher in “imagery use during practice and competition” and socioeconomic status in English soccer academy athletes (*p* = 0.55). Hauw et al. (53) noted Swiss soccer academy players scored within the range consistent with good developers. Hill et al. (55) found the use of “imagery and active preparation” (PCDEQ—Version 2 Factor 2) had no significant effect (*p* = > 0.05) on being rated by coaches to progress to the top level in their sport. However, Barraclough et al. (56) demonstrated “imagery and active preparation” (PCDEQ—Version 2 Factor 2) had a significant large effect on becoming a category 1 player (*d* = 1.00, *p* = <0.001). With smaller effects found between levels of participation (category levels) (*d* = 0.32, *p* = < 0.009) and age groups (*d* = 0.48, *p* = < 0.001).

Erikstad et al. (68) found Norwegian soccer players in a national talent development program who scored high in self-regulation were significantly more likely to be selected at a national level compared to those players who scored lower on self-regulation (*p* = <0.05). Cumming et al. (69) found a small statistically significant correlation between biological maturity and self-regulation use (*r* = −0.17, *p* = < 0.05) in a cohort of English soccer academy players.

“Evaluating performance and working on weaknesses” (PCDE—version 1, factor 5) scores appeared to show age-related changes, that differed based on eventual scholarship status for English academy soccer players (6). Scores increased with age for those players who went on to hold category 1 and 2 scholarships compared to those who didn’t receive a scholarship (*p* = < 0.05). Kelly et al. (52) found no significant change in English academy soccer players who scored differently in this factor from those playing up an age, compared to those who didn’t (*p* = 0.37). Kelly et al. (54) found no significant differences between “higher” or “lower” potential athlete scores for “evaluating performances and working on weaknesses” and socioeconomic status in English soccer academy athletes (*p* = 0.55). Hauw et al. (53) noted Swiss soccer academy players scored within the range to be considered “good developers” (*m* = 5.07, SD = 0.60).

“Self-directed control and management” (PCDEQ—Version 2 Factor 2) was found by Hill et al. (55) to have a medium effect (*d* = 0.07, *p* = <0.001) on the likelihood of players progressing to the top level of their sport (as rated by the coach) across, soccer, rugby league and rugby union, when compared to the coaches ratings of players having a low likelihood of progression. Barraclough et al. (56) found significant differences between age groups and levels of participation for the use of self-directed control and management (*p* = 0.002) and (*p* = <0.001).

Figure 3 provides a visual map of the ICF model with significant relationships between mental functions and academy participation as well as interactions with environmental and personal factors according to available published literature identified in this review. Table 12 provides further insights into mapped ICF categories, sub-categories, for mental functions (psychological attributes) and the documented relationships with activity and participation (for academy athletes) as well as the relationships with environmental and personal contextual factors.

**Table 12:** ICF framework, mapping ICF sub-categories and mental functions to academy activities and participation, including contextual factors.

Mental Functions	Activities & Participation		Contextual factors (Environmental and Personal)	
Orientation functions				
Orientation to self (total athletic identity) (2, 49)	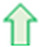	Discriminates between soccer academy athletes and school peers (2).		
Global Psychosocial functions				
Global Psychosocial *(*Seeking and using social support) (55, 56)	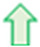	Higher likelihood of progressing to the top level of their sport (55).		
Temperament and personality functions				
Conscientiousness (perfectionistic tendencies) (55, 56)	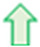	Higher likelihood of progressing to the top level of their sport (55). Higher soccer category/scholarship status achieved (56).	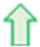	Increased with age (56).
Energy and drive functions				
Energy level (subjective vitality) (57, 58)			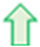	Perceived autonomy support form coach (57) and perceived basic psychological needs satisfaction from the coach were achieved (58).
Motivation (task orientation) (50, 59–62)	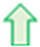	Discriminates between elite vs. non-elite academy soccer players (59, 60).		
Motivation (ego orientation) (50, 59–62)			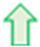	Scores significantly differentiated between under-12 and under-17 age groups (62).
Motivation (win orientation) (62–64)	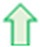	Increased retention into regional academy squad 12-month later (64).	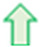	Scores significantly differentiated between under-12 and under-17 age groups (62).
Motivation (goal orientation) (62–65)	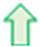	Increased retention into regional academy squad 12-month later (64).		
Motivation (motivation) (50, 51)	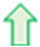	Discriminate between elite and non-elite youth field hockey athletes (51).		
Motivation (competitiveness) (62, 63)	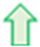	Greater likelihood of promotion to professional soccer status (62).		
Motivation (hope for success) (62, 66)	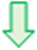	Greater likelihood of promotion to professional soccer status (62).		
Motivation (fear of failure) (62, 66)	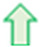	Greater likelihood of promotion to professional soccer status (62).		
Motivation (self-determination) (64, 65)	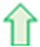	Increased retention into regional academy squad 12-month later (64).		
Energy and drive functions, other specified, mental health (clinical indicators) (55, 56)	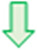	Higher likelihood of progressing to the top level of their sport (55). Higher soccer category/scholarship status achieved (56).		
Global mental functions				
Mental health (exhaustion) (57, 58, 67)			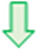	Perceived autonomy support form coach (57). Perceived basic psychological needs satisfaction from the coach were achieved (58).
Mental health (burnout) (57, 58, 67)			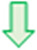	Perceived autonomy support form coach (57). Perceived basic psychological needs satisfaction from the coach were achieved (58, 67).
Emotional functions				
Regulation of emotion (coping with performance and developmental pressures) (6, 52–54)	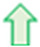	Athletes would achieve higher soccer scholarship status outcomes (6). High-potential soccer athlete (54).	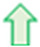	Increased with age (6). Athletes from lower socioeconomic status (54).
Regulation of emotion (adverse response to failure) (55, 56)	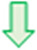	Higher likelihood of progressing to the top level of their sport (55).	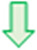	Increased with age (56).
Regulation of emotion (Active coping) (55, 56)	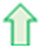	Higher soccer category/scholarship status achieved (56). Higher likelihood of progressing to the top level of their sport (55).		
Higher-level cognitive function				
Organisation and planning (Imagery use during practice and competition) (6, 52, 53)			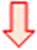	Decreased use with age in category 2 youth soccer academy athletes (6).
Organisation and planning (Imagery and active preparation) (55, 56)	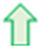	Highest use within category one, youth academy athletes (56).	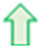	Higher scores were seen in older age youth soccer academy athletes (56).
Higher-level cognitive function (Self-regulation) (68, 69)	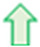	National soccer team selection (68).	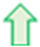	Biological maturation, late maturing athletes increased use of self-regulation (69).
Higher-level cognitive function (Evaluating performance and working on weaknesses) (6, 52–54)			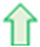	Scores showed age related changes, with older players scoring highest (6).
Higher-level cognitive function (self-directed control and management) (55, 56)	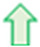	Higher likelihood of progressing to the top level of their sport (55). Higher soccer category/scholarship status achieved (56).		

Table 1, key: 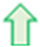 high levels positively discriminate. 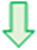 Low levels positively discriminate. 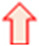 High levels negatively discriminate. 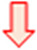 Low levels negatively discriminate.

## 4 Discussion

The primary aim of this systematic review was to profile mental functions of male youth team-based ball-sport athletes in academy-based programs and to explore the interactions between mental functions and activity/participation outcomes and environmental and personal contextual factors via an ICF lens. One hundred and seventy-eight mental functions were identified across 12 ICF categories. A meta-aggregation was possible for thirty-two mental functions across eight ICF categories. The eight categories included orientation, global psychosocial, temperament and personality, energy and drive, global mental functions, attention, emotion and high-level cognitive functions. Meta-aggregated results (Tables 4–11) revealed male youth team-based ball-sport academy athletes displayed; moderate to high levels of psychosocial function and conscientiousness, and high confidence levels; moderate to high levels of energy and drive functions, moderate levels of attention, and organisation and planning functions with moderate to high levels of self- and emotion-regulation. Further, these athletes demonstrated low levels of mental health issues, and low levels of exhaustion and burnout.

From the eight categories of mental functions, seven showed significant associations with other ICF domains. As highlighted in Figure 3, the seven mental functions; orientation, temperament and personality, energy and drive, global mental functions, mental health, emotional functions, and high-level cognitive functions and the subsequent 14 mental functions have been mapped to other domains within the ICF framework. The interactions are illustrated between mental functions and other domains such as body structure, activity, participation, environmental and personal factors, highlighting the biopsychosocial holistic approach to viewing talented athletes. By mapping biopsychosocial athlete attributes via the ICF framework the potential contribution that mental functions have on future activities and participation levels of male youth team-based ball-sport athletes in academy programs becomes evident. The findings from this systematic review and meta-aggregation outline possible attributes that could be further explored in male youth team-based ball-sport athletes to successfully navigate through the academy system. However, the development and contribution of these attributes will need to be explored through future intervention-based research. Additionally, in some cases this ICF mapping process also shows how academy-based activities and participation levels can positively or negatively interact with the athletes’ mental functions. The findings from this systematic review should be considered by those who seek to best develop academy based male athletes in team-based ball-sports and those planning further research on this topic.

### 4.1 Orientation functions

Total athletic identity scores were significantly larger for English academy soccer players when compared to school peers (2). This finding would be considered consistent as behaviours exhibited by high scoring players for this mental function have advantages of increased self-esteem, positive body image, and work ethic (2). Identity theory proposes that identity (i.e., “I’m an athlete”) leads to behaviour choices and habits that match the expectations to that identity (70). As a result, to meet the demands of academy programs, players need to perform habits that can lead to success along the talent pathway, that align to their “athletic identity”. Alternatively, negative consequences of high athletic identity can be associated with both short- and long-term effects. Rongen et al. (2) suggests negative consequences may include an inability to cope with obstacles (i.e., deselection or injury), overtraining and burnout, risk one's own health, less focus on education, and delayed career development, as well as being unprepared to transition out of sport that results in identity loss, which may lead to mental health issues such as decreased well-being and depression. It is therefore important that sporting academies focus on how programs are designed, implemented, and managed to enhance facilitation of positive psychosocial outcomes by actively monitoring mental functions such as athlete identity and activating early intervention if needed to prevent negative long-term consequences for athletes.

### 4.2 Temperament and personality functions

Findings by Barraclough et al. (56) suggest perfectionistic tendencies may increase with age and level of sport participation with the highest perfectionistic behaviours evident in category 1 players (highest level of participation within the academy) who were older. This multidimensional effect is seen as either perfectionistic striving, which indicates one's determination for excellence, self-organisation (i.e., routines and structures), and setting high personal performance standards (71). As opposed to perfectionistic concerns which relates to fears from previous mistakes and social evaluations, as well as imbalances between expectations and performance (56, 71). A previously published review by Hill et al. (72) noted that perfectionism may change across the lifespan due to developmental changes, with the possibility of younger players displaying a naive level of optimism. Barraclough et al. (56) suggested the importance of winning and setting outcome-based goals, such as selection into the next phase of the academy program may increase throughout adolescence which could drive increasing levels of perfectionism. Jordana et al. (71) describes this phenomenon as the “perfectionism paradox” where high performance sport demands a perfectionistic approach, and players who are overly concerned about achieving such a level of perfectionism can be susceptible to negative consequences such as a lack of motivation, ill-being and ultimately a decrease in performance (71). Therefore, providing focused training for athletes on ways to avoid the negative consequences of perfectionism, should form a pivotal part of academy player wellbeing programs (56) and opportunity to develop dual careers (i.e., academic and athletic) favouring a holistic athlete development process should be prioritised in academy programs, ensuring a “plan B” for players (71).

### 4.3 Energy and drive functions

Two studies highlighted the importance of “perceived coach autonomy support” and its positive impact on energy levels (subjective vitality) for academy youth soccer players (57, 58). Findings from these studies highlight the importance of nurturing feelings of autonomy and competence in youth academy athletes to enhance energy and drive. Academy coaches who provide autonomy support and promote a sense of competence and autonomy, may help contribute to the improved well-being of academy players. This notion is supported by the basic needs theory proposed by, Ryan and Deci (73) which suggests positive growth and development in humans occurs by creating an energised state, which is advanced by meeting three inherent psychological needs; feelings of competence, autonomy and relatedness, and if these needs are not nurtured (for example by coaches in academy programs) they may contribute to pathology and ill-being for athletes.

Kavussanu et al. (59) and Reilly et al. (60) found significant differences in motivation (task orientation) between levels of elite vs. non-elite soccer academy players. Suggesting players within elite academy programs may be more focused on personal improvement, learning and effort, which are characteristics of task orientated individuals (59, 74). However, Bennett et al. (61) and Huijgen et al. (50) both found no significant differences in task orientation within academy programs, regardless of their tier or selection status. These results may indicate that once academy players are within a structured environment, the differences in task orientation that were observed between elite and non-elite players may reduce, and highlights opportunity for academies to promote uniform emphasis on personal improvement and effort among their players, irrespective of their specific tier or selection status.

Joseph et al. (63) revealed that selected and non-selected under-16 and under-18 basketball talent pathway athletes exhibited no significant differences in win orientation scores. Opposingly, the findings by Zuber et al. (64) indicated that win-oriented, failure-fearing soccer players, while not selected into the National under-15s squad, were more likely to remain in regional squads a year later. This suggests that for some sports, a win-oriented mindset might not be a decisive factor in immediate selection to national-level squads but may contribute to player retention in a talent development pathway. Wachsmuth et al. (62) found German soccer academy athletes who scored high in competitiveness, high in hope for success but low fear of failure significantly increased their odds of progression in professional soccer status.

### 4.4 Global mental functions, mental health

Hill et al. (55) observed that, among UK academy-based players, those who were perceived by their coaches as having a higher likelihood of advancing to the elite level exhibited significantly lower scores in clinical indicators (PCDEQ—Version 2 Factor 7) compared to other players, who were deemed less likely to reach the highest levels of their sport. This finding suggests that players with lower clinical indicator scores may possess certain mental functions or characteristics that are conducive to their coaches’ expectations of future success. Furthermore, Barraclough et al. (56) investigated clinical indicator scores across different levels of youth soccer academy participation, revealing significant differences among various participation categories. However, age, did not significantly influence these scores, implying that clinical indicators of global mental functions, while associated with the level of participation, might not necessarily change significantly as players progress through different age groups within an academy structure.

Adie et al. (57) found that levels of exhaustion were significantly different among elite youth soccer academy players, with lower autonomy support from coaches linked to higher emotional and physical exhaustion. While no significant within-person relationships emerged between basic psychological needs and exhaustion, the interaction between relatedness to others and how exhaustion changed over time played a role in predicting exhaustion levels. Curran et al. (67) expanded upon this by showing that when psychological needs of youth soccer players in the UK were met, they were less likely to experience emotional and physical exhaustion, underscoring the importance of psychological well-being in preventing burnout. This supports findings from Cheval et al. (58), who found that coach autonomy support, as well as satisfaction or frustration of the need for competence and autonomy, were significant predictors of burnout among French youth soccer players. Furthermore, Curran et al. (67) identified a small but significant correlation between harmonious passion and burnout among youth soccer players in the UK. These collective results suggest at the individual level, satisfaction or frustration with meeting psychological needs contributes to the likelihood of experiencing burnout, and psychological needs should be monitored and nurtured through the academy programs to prevent exhaustion and burnout.

### 4.5 Emotional functions

Coping with performance, and developmental pressure scores, reported by Saward et al. (6) reveals increases in coping scores with age, highlighting the potential for psychological development and adaptation as players progress through their academy pathway. Players who eventually received higher-level scholarships scored significantly higher in this factor, suggesting the importance of effective coping strategies in securing advanced opportunities within academy programs (Saward et al, 2019). Additionally, Kelly et al. (52) found that the ability to cope with these pressures did not significantly differ between academy soccer players who played up an age group and those who did not, further emphasising the complexity of the relationship between coping abilities and development within the academy context.

Whilst examining adverse response to failure scores, Hill et al. (55) demonstrated that youth academy players whose coaches believed they were more likely to advance to the elite level in their sport reported significantly lower adverse response to failure behaviours. These findings may suggest that the athletes who were seen as having greater potential might perceive a greater ability to respond positively to setbacks experienced during the academy pathway such as injury or deselection. Barraclough et al. (56) found significant effects related to age and levels of participation within youth soccer academy players, where older players, particularly in the under-16 and youth soccer teams, reported the highest scores in adverse response to failure behaviours, emphasising the potential developmental challenges faced by players as they advance in age through academy programs. Additionally, the higher scores in category 1 soccer academies, suggests that the elite level of play may introduce additional opportunities for development related to reducing adverse responses to failure, supporting athletes to develop the skill set required to manage adverse responses to failure and better equipping them for future setbacks. As academy players progress through the pathway there is an increased chance of adverse response to failure which may coincide with mores stressful challenges and transitions as players progress to higher levels of participation (56).

The findings from Barraclough et al. (56) and Hill et al. (55) collectively provide an understanding of active coping behaviours and mechanisms in the context of youth academy team-based ball-sport athletes. Barraclough et al. (56) also found small but significant differences in active coping behaviours across top-tier academy categories. Whereas, Hill et al. (55) found athletes whose coaches believed they were more likely to reach the elite level in their respective sports reported significantly higher scores in active coping behaviours compared to those perceived as having a lower likelihood of success. This indicates that athletes with higher potential may exhibit more proactive approaches and mental skills to manage stress and challenges. These results collectively underscore the importance of considering the competitive context and the influence of coach expectations on coping behaviours, with potential implications for athlete development and performance in youth sports.

### 4.6 High-level cognitive functions

Studies exploring high-level cognitive functions (6, 52, 53, 55, 56), presented varying views regarding the impact of utilising imagery during practice and competition in the context of youth soccer players. The collective findings across these studies demonstrates imagery and active preparation is frequently used by players in higher categories of participation within academy programs.

When exploring the relationship between self-regulation and participation in youth soccer academies, Erikstad et al. (68) demonstrated a significant link between high levels of self-regulation and the likelihood of being selected at a national level within a Norwegian talent development program. It is possible that players who exhibit effective self-regulation skills may be better equipped to handle the demands of high-level competition and, consequently, are more likely to progress to the national level. Cumming et al. (69) extended this understanding by revealing late maturing soccer players utilised self-regulation skills more than early or average maturing players, highlighting the opportunities to development these skills with athletes along the academy pathway.

The findings by Saward et al. (6), Kelly et al. (52), Hauw et al. (53), offer insights into the dynamics of academy athletes evaluating performance and working on weaknesses. Self-assessment and improvement can be seen as a desirable mental function, as it indicates a proactive approach to skill development and performance enhancement (75). Hauw et al. (53) noted that Swiss soccer academy players scored within the range indicative of good developers in the domain of evaluating performance and working on weaknesses. Saward et al. (6) demonstrated age-related changes in this mental function, particularly among English academy soccer players who eventually secured category 1 and 2 scholarships. Kelly et al. (52) however, found no significant change in this mental function among English academy soccer players who played up an age level compared to those who did not, suggesting the act of playing against older peers did not significantly influence the utilisation of evaluating performance and weaknesses. These findings demonstrate the importance of proactive self-assessment among youth academy players. The ability to recognise areas of improvement and actively address weaknesses can play a key role in their development and eventual success. Talent development programs should consider strategies for encouraging and supporting these behaviours, particularly as players advance in age and skill level. This not only contributes to their individual growth but can also have positive implications for the overall quality of talent within academy programs (6, 75).

Studies by Hill et al. (55) and Barraclough et al. (56) provide valuable insights into the role of self-directed control and management in talent academies within youth sports. Particularly in the context of soccer, rugby league, and rugby union athletes who exhibited high levels of self-directed control and management, were more likely to be rated (by their coach) as having the potential to progress to the top level of their respective sports (55). This indicates the vital role of self-regulation and the ability to independently manage one's actions and decisions in the context of talent development. Barraclough et al. (56) findings further contribute to this understanding by revealing significant differences in the use of self-directed control and management based on age groups and levels of participation. These differences underscore the dynamic nature of self-regulation skills which require focused attention for development as players progress through different age categories and participation levels within academy programs.

The secondary aim of this review was to identify the psychometric tools used to assess mental functions of male youth team-based ball-sport athletes. A variety of tools (*n* = 66) were used across all 178 mental functions, with no definitive tool used to capture this data. Hill et al. (55) suggest the use of a triangular approach using a psychometric tool, direct observations, and interviews to assess mental functions. However, this may be problematic for academies with limited resources (personnel and financial). The most used tool to assess mental functions in the male youth team-based ball-sport academy setting was the Task and Ego Orientation in Sport questionnaire (*n* = 5) as a measure to assess energy and drive functions. Whilst the Psychological Characteristics of Developing Excellence—version 1 questionnaire (PCDEQ1) (*n* = 4) was most used to assess a variety of mental functions. An updated version of the PCDEQ1, Psychological Characteristics of Developing Excellence—version 2 (PCDEQ2) is available and was identified by two studies within this systematic review. The PCDEQ2 offers sports practitioners working with youth athletes, a cost-effective age-appropriate tool to assess multiple mental functions across a variety of categories and sub-categories including; global psychosocial, temperament and personality, energy and drive (clinical indicators), emotional and high-level cognitive functions.

### 4.7 Limitations

Limitations of this systematic review were that studies were excluded if they did not specifically state participants were in a talent development or academy-based program. This may have led to relevant studies being excluded if they had ambiguous participant details. This could also include studies from countries where talent academy programs do not exist, instead talented athletes are developed through school-based systems. A population bias exists where 42 out of the 50 studies for this review involved soccer academies, with 92% coming from the UK and European nations, limiting the generalisability of the findings to all academy-based sports.

The lack of longitudinal studies identified in this systematic review highlights the need for application of longitudinal investigations (28). Such a methodological approach could be utilised to profile the rate of change in mental functions over time for talented youth male ball-sport athletes. Such research could lead to identifying mental functions that remain stable or those that are more modifiable over time, and that information could guide interventions utilised in the talent academy pathway programs to enhance participation outcomes for athletes.

The limited number of studies using the same psychometric tool, to assess mental functions, increases the potential impact of bias (e.g., publication and response bias) for the meta-aggregated findings and subsequent conclusions in this review. However, studies appear to be homogenous as identified by lower standard deviation scores and the participant demographics (i.e., soccer Academy from UK). Future research could utilise the findings from this research to utilise consistent tools when evaluating mental functions to develop larger powered meta-aggregated findings with addition of athletes from team-based ball-sports outside the UK and European nations. A response bias may exist amongst the literature as when academy participants are responding to questionnaires, participants may give untruthful or favourable responses as to appear a certain way for the coach, sport practitioners, medical staff, or their peers, as well as their own perceptions of what will influence their selection or outcomes (76).

### 4.8 Conclusions

This paper explores the psychological attributes of male youth team-based ball-sport academy athletes and demonstrated that this cohort present with low levels of clinical indicators and burnout, moderate levels of psychosocial functions, conscientiousness, regulations of emotion, and organising and planning, moderate to high levels of motivation and self-regulation, with high levels of energy and orientation to self. Many of these mental functions have been associated with successful outcomes such as academy selection and promotion to the elite level of participation.

The plethora of mental functions examined, and the varied use of tools highlights the complexities in the sport psychology literature and their multifaceted interactions with other domains within the talent development pathway. This review demonstrates a practical framework that can capture these complex biopsychosocial interactions using a common language; the ICF, developed by the WHO, which could be a useful tool for sports practitioners to provide an individualised and holistic approach to the development of youth athletes in talent academy-based programs.

## Data Availability

The original contributions presented in the study are included in the article/Supplementary Material, further inquiries can be directed to the corresponding author.

## Supplementary material

Supplementary Table S1 Excluded studies from full text.

Supplementary Table S2 Critical appraisal scores.

Supplementary Table S3 Data extraction Table.

Supplementary Table S4 Mapped mental functions.

Supplementary Table S5 Meta-aggregated data.
