# Supplementary material for: Exploring mental functions utilised by male youth team-based ball-sport athletes within academy programs: a systematic review and meta-aggregation
Source: Front Sports Act Living. 2024 Oct 17;6:1453817. doi: 10.3389/fspor.2024.1453817 (PMC11524844; doi:10.3389/fspor.2024.1453817)
Supplement: Supplementary Table S4 — Mapped mental functions. [file Table4.docx]

**Supplementary file 4.** Mental functions (i.e. psychological attributes) mapped according to ICF classifications and tools used to measure each mental functions.

| **Measured mental functions**  **(psychological attributes) from studies** | **ICF-CY category** | **ICF-CY sub-category** | **Tools** | **Author/s (year)** |
| --- | --- | --- | --- | --- |
| Flow | **b110 Consciousness functions** | **b 1108 Consciousness functions, other specified, flow** | Flow State Scale | Bakker et al., (2011) |
| Total athletic identity | **b114 Orientation functions** | **b11420 Orientation to self** | Athletic Identity Measurement Scale | Mitchell et al., (2014)  Rogen et al., (2020) |
| Social identity (view themselves as an athlete) |  |  |  | Mitchell et al., (2014)  Rogen et al., (2020) |
| Exclusivity |  |  |  | Mitchell et al., (2014)  Rogen et al., (2020) |
| Negative affectivity |  |  |  | Mitchell et al., (2014)  Rogen et al., (2020) |
| Social identity |  | **b11421 Orientation to others** | Social Identification Questionnaire | Barker et al., (2014) |
| Friendship identity content |  |  |  |  |
| Results identity content |  |  |  |  |
| Collective efficacy |  |  | Collective Efficacy Questionnaire |  |
| Team emphasis |  |  | Psychological Skills Inventory for Sports | Elferink-Gemser et al., (2004)  Elferink-Gemser et al., (2007)  Huijgen et al., (2014) |
| Communication | **b122 Global psychosocial functions** |  | 5C questionnaire | Harwood et al., (2015) |
| Support for long term success |  |  | Psychological Characteristics of Developing Excellence Questionnaire | Saward et al., (2019)  Kelly et al., (2020)  Kelly et al., (2023)  Hauw et al., (2022) |
| Seeking and using social support |  |  | Psychological Characteristics of Developing Excellence Questionnaire-version 2 | Hill et al., (2019)  Barraclough et al. (2022) |
| Team orientation |  |  | Combined observations and questionnaires | Bolckmans et al. (2023) |
| Confidence (interpersonal) |  |  | Mental Toughness Questionnaire-48 | Tredrea et al., (2017) |
| Coachability |  |  | Athletic Coping Skills Inventory Coping Skills-28 | Csáki et al., (2017) |
| Effort | **b125 Dispositions and intra-personal functions** | **b1252 Activity level** | Self-Regulation of Learning Self-Report Scale | Toering et al., (2012) |
| Commitment |  | **b1254 Persistence** | 5C questionnaire | Harwood et al., (2015) |
| Executing control to express affection |  | **b1255 Approachability** | Coping functions and behaviours tool | Rumbold et al., (2020) |
| Extraversion | **b126 Temperament and personality functions** | **b1260 Extraversion** | Revised NEO personality inventory | Rumbold et al., (2020) |
|  |  |  | Personality for Professional Inventory (French version) | Hauw et al., (2022) |
|  |  |  | International Personality Item Pool | McAuley et al., (2022) |
| Enthusiasm |  |  | Personality for Professional Inventory (French version) | Hauw et al., (2022) |
| Sociability |  |  | Personality for Professional Inventory (French version) | Hauw et al., (2022) |
| Energy |  |  | Personality for Professional Inventory (French version) | Hauw et al., (2022) |
| Assertiveness |  |  | Personality for Professional Inventory (French version) | Hauw et al., (2022) |
| Agreeableness |  | **b1261 Agreeableness** | Personality for Professional Inventory (French version) | Hauw et al., (2022) |
|  |  |  | International Personality Item Pool | McAuley et al., (2022) |
| Competitiveness |  |  | Personality for Professional Inventory (French version) | Hauw et al., (2022) |
| Orientation toward others |  |  | Personality for Professional Inventory (French version) | Hauw et al., (2022) |
| Trust |  |  | Personality for Professional Inventory (French version) | Hauw et al., (2022) |
| Compliance |  |  | Personality for Professional Inventory (French version) | Hauw et al., (2022) |
| Ability to organise and engage in quality practice |  | **b1262 Conscientiousness** | Psychological Characteristics for developing excellence | Saward et al., (2019)  Kelly et al., (2020)  Kelly et al., (2023)  Hauw et al., (2022) |
| Conscientiousness |  |  | Personality for Professional Inventory (French version) | Hauw et al., (2022) |
|  |  |  | International Personality Item Pool | McAuley et al., (2022) |
| Organisation |  |  | Personality for Professional Inventory (French version) | Hauw et al., (2022) |
| Self-discipline |  |  | Personality for Professional Inventory (French version) | Hauw et al., (2022) |
| Impulse control |  |  | Personality for Professional Inventory (French version) | Hauw et al., (2022) |
| Achievement striving |  |  | Personality for Professional Inventory (French version) | Hauw et al., (2022) |
| Perfectionistic tendencies |  |  | Psychological Characteristics of Developing Excellence Questionnaire-version 2 | Hill et al., (2019)  Barraclough et al. (2022) |
| Personal standards |  |  | Sport Multidimensional Perfectionism Scale -2 | Jordana et al., (2022) |
| Concern over mistakes |  |  | Sport Multidimensional Perfectionism Scale -2 | Jordana et al., (2022) |
| Perceived parental pressure |  |  | Sport Multidimensional Perfectionism Scale -2 | Jordana et al., (2022) |
| Perceived coach pressure |  |  | Sport Multidimensional Perfectionism Scale -2 | Jordana et al., (2022) |
| Self-organisation |  |  | Sport Multidimensional Perfectionism Scale -2 | Jordana et al., (2022) |
| Emotional stability |  | **b1263 Psychic stability** | Personality for Professional Inventory (French version) | Hauw et al., (2022) |
| Sensitiveness |  |  | Personality for Professional Inventory (French version) | Hauw et al., (2022) |
| Self-confidence |  |  | Personality for Professional Inventory (French version) | Hauw et al., (2022) |
| Stress vulnerability |  |  | Personality for Professional Inventory (French version) | Hauw et al., (2022) |
| Frustration tolerance |  |  | Personality for Professional Inventory (French version) | Hauw et al., (2022) |
| Neuroticism |  |  | Revised NEO personality inventory | Rumbold et al., (2020) |
|  |  |  | International Personality Item Pool | McAuley et al., (2022) |
| Demandingness |  |  | Irrational Performance Belief Inventory | Jordana et al., (2022) |
| Low frustration tolerance |  |  | Irrational Performance Belief Inventory | Jordana et al., (2022) |
| Awfulizing |  |  | Irrational Performance Belief Inventory | Jordana et al., (2022) |
| Depreciation |  |  | Irrational Performance Belief Inventory | Jordana et al., (2022) |
| Openness to experience |  | **b1264 Openness to experience** | Personality for Professional Inventory (French version) | Hauw et al., (2022) |
|  |  |  | International Personality Item Pool | McAuley et al., (2022) |
| Innovativeness and creativity |  |  | Personality for Professional Inventory (French version) | Hauw et al., (2022) |
| Intellectual versus action orientation |  |  | Personality for Professional Inventory (French version) | Hauw et al., (2022) |
| Self-observation |  |  | Personality for Professional Inventory (French version) | Hauw et al., (2022) |
| Openness to change |  |  | Personality for Professional Inventory (French version) | Hauw et al., (2022) |
| Self-esteem |  | **b1266 Confidence** | Self-description Questionnaire-2 | Cheval et al., (2017) |
| Confidence |  |  | Athletic Coping Skills Inventory Coping Skills-28 | Csáki et al., (2017) |
|  |  |  | Psychological Skills Inventory for Sports | Elferink-Gemser et al., (2004)  Elferink-Gemser et al., (2007)  Huijgen et al., (2014) |
|  |  |  | 5C questionnaire | Harwood et al., (2015) |
| Self-confidence |  |  | Combined observations and questionnaires | Bolckmans et al. (2023) |
| Confidence (abilities) |  |  | Mental Toughness Questionnaire-48 | Tredrea et al., (2017) |
| Self-efficacy |  |  | Self-Regulation of Learning Self-Report Scale | Toering et al., (2012) |
|  |  |  | Self-efficacy scale | Turner et al., (2013) |
|  |  |  | Self-efficacy in soccer | Feichtinger & Honer (2015)  Wachsmuth et al., (2023) |
| General self-concept |  |  | Physical self-concept scales | Feichtinger & Honer (2015)  Wachsmuth et al., (2023) |
| Specific self-concept |  |  |  |  |
| Loss of confidence |  |  | General Health Questionnaire-12 | Ivarsson et al., (2015)  Jordana et al., (2022) |
| Subjective vitality | **b130 Energy and drive functions** | **b1300 Energy level** | Subjective Vitality Scale | Adie et al., (2012)  Cheval et al., (2017) |
| Autonomy |  | **b1301 Motivation** | Most satisfying event questionnaire | Adie et al., (2012) |
|  |  |  | Job Content Instrument | Bakker et al., (2011) |
|  |  |  | Needs Satisfaction Thwarting Scale | Cheval et al., (2017) |
|  |  |  | Autonomy scale | Curran et al., (2013) |
|  |  |  | Balanced Measure of Psychological Needs | Rogen et al., (2020) |
| Competence |  |  | Intrinsic Motivation Inventory | Adie et al., (2012) |
|  |  |  |  | Curran et al., (2013) |
|  |  |  | Job Content Instrument | Bakker et al., (2011) |
|  |  |  | Needs Satisfaction Thwarting Scale | Cheval et al., (2017) |
|  |  |  | Balanced Measure of Psychological Needs | Rogen et al., (2020) |
| Relatedness |  |  | Need for Relatedness Scale | Adie et al., (2012) |
|  |  |  | Needs Satisfaction Thwarting Scale | Cheval et al., (2017) |
|  |  |  | Acceptance Scale | Curran et al., (2013) |
|  |  |  | Balanced Measure of Psychological Needs | Rogen et al., (2020) |
| Task orientation |  |  | Task and Ego Orientation in Sport Questionnaire | Bennett et al., (2020)  Huijgen et al., (2014)  Kavussanu et al., (2011)  Reilly et al., (2000)  Wachsmuth et al., (2023) |
|  |  |  | Perceived Motivational Climate in Sport Questionnaire-2 | Csáki et al., (2017) |
| Hope for success |  |  | Achievement Motive Scale-Sport | Feichtinger & Honer (2015)  Wachsmuth et al., (2023)  Zuber et al., (2014)  Zuber et al., (2015)  Zuber et al., (2016) |
| Competitiveness |  |  | Sport Orientation Questionnaire | Joseph et al., (2021)  Wachsmuth et al., (2023) |
| Win Orientation |  |  | Sport Orientation Questionnaire | Joseph et al., (2021)  Wachsmuth et al., (2023)  Zuber et al., (2015) |
| Ego orientation |  |  | Task and Ego Orientation in Sport Questionnaire | Bennett et al., (2020)  Huijgen et al., (2014)  Kavussanu et al., (2011)  Reilly et al., (2000)  Wachsmuth et al., (2023) |
|  |  |  | Perceived Motivational Climate in Sport Questionnaire-2 | Csáki et al., (2017) |
| Fear of failure |  |  | Achievement Motives Scale-Sport | Feichtinger & Honer (2015)  Wachsmuth et al., (2023)  Zuber et al., (2014)  Zuber et al., (2015) |
| Experiencing shame and embarrassment |  |  | Performance Failure Appraisal Inventory | Sagar et al., (2010) |
| Having an uncertain future |  |  |  |  |
| Upsetting important others |  |  |  |  |
| Important others losing interest |  |  |  |  |
| Devaluing self-estimate |  |  |  |  |
| Performance approach goals |  |  | Achievement Goals Questionnaire | Turner et al., (2013) |
| Performance avoidance goals |  |  |  |  |
| Mastery approach goals |  |  |  |  |
| Mastery avoidance goals |  |  |  |  |
| Achievement motivation |  |  | Achievement Motives Scale-Sport | Schorer et al., (2010)  Sieghartsleitner et al., (2019) |
| Winning mindset |  |  | Combined observations and questionnaires | Bolckmans et al. (2023) |
| Goal Orientation |  |  | Sport Orientation Questionnaire | Joseph et al., (2021)  Sieghartsleitner et al., (2019)  Wachsmuth et al., (2023)  Zuber et al., (2015) |
| Amotivation |  |  | Sport Motivation Scale | Csáki et al., (2017) |
|  |  |  | Sport Motivation Scale-II | Ronald et al., (2018) |
|  |  |  | Behavioural Regulation in Sport Questionnaire | Hendry et al., (2019) |
| Extrinsic motivation |  |  | Sport Motivation Scale | Csáki et al., (2017) |
|  |  |  | Sport Motivation Scale-II | Ronald et al., (2018) |
|  |  |  | Behavioral Regulation in Sport Questionnaire | Hendry et al., (2014)  Hendry et al., (2019) |
| Introjected regulation |  |  | Behavioral Regulation in Sport Questionnaire | Hendry et al., (2014)  Hendry et al., (2019) |
|  |  |  | Sport Motivation Scale-II | Ronald et al., (2018) |
| Identified regulation |  |  | Behavioral Regulation in Sport Questionnaire | Hendry et al., (2014)  Hendry et al., (2019) |
|  |  |  | Sport Motivation Scale-II | Ronald et al., (2018) |
| Integrated regulation |  |  | Behavioral Regulation in Sport Questionnaire | Hendry et al., (2014)  Hendry et al., (2019) |
|  |  |  | Sport Motivation Scale-II | Ronald et al., (2018) |
| Intrinsic motivation |  |  | Sport Motivation Scale | Csáki et al., (2017) |
|  |  |  | Sport Motivation Scale-II | Ronald et al., (2018) |
|  |  |  | Behavioral Regulation in Sport Questionnaire | Hendry et al., (2014)  Hendry et al., (2019) |
| Motivation |  |  | Psychological Skills Inventory for Sports | Elferink-Gemser et al., (2004)  Elferink-Gemser et al., (2006)  Elferink-Gemser et al., (2007)  Huijgen et al., (2014) |
| Self-determination index |  |  | Behavioral Regulation in Sport Questionnaire | Hendry et al., (2014)  Hendry et al., (2019) |
|  |  |  | Sport Orientation Questionnaire | Sieghartsleitner et al., (2019)  Zuber et al., (2015) |
| Determination |  |  | Psychological Performance Inventory - Alternative | Joseph et al., (2021) |
| Autonomous motivation |  |  | Behavioral Regulation in Sport Questionnaire | Hendry et al., (2014)  Hendry et al., (2019) |
| Controlled motivation |  |  | Behavioral Regulation in Sport Questionnaire | Hendry et al., (2014)  Hendry et al., (2019) |
| Compassion (Intention) |  |  | Compassion Motivation and Action Scales | Beavan et al., (2022) |
| Compassion (Distress tolerance) |  |  |  |  |
| Compassion (Action) |  |  |  |  |
| Self-compassion (Intention) |  |  |  |  |
| Self-compassion (Distress tolerance) |  |  |  |  |
| Self-compassion (Action) |  |  |  |  |
| Harmonious passion |  |  | Passion for sport scale | Curran et al., (2013) |
| Obsessive passion |  |  |  |  |
| Clinical indicators  (combines items of mental health) |  | **b1308 Energy and drive functions, other specified, mental health** | Psychological Characteristics of Developing Excellence Questionnaire-version 2 | Hill et al., (2019)  Barraclough et al. (2022) |
| Mental toughness | **b139 Global mental functions, others specified and unspecified.** | **b1390 Global mental functions, others specified, mental toughness** | Mental Toughness Questionnaire-18 | Crust., (2010) |
|  |  |  | Mental Toughness Questionnaire-48 | Tredrea et al., (2017) |
|  |  |  | Mental Toughness Index | McAuley et al., (2022) |
| Determination |  |  | Psychological Performance Inventory - Alternative | Joseph et al., (2021) |
| Self-belief |  |  | Psychological Performance Inventory - Alternative | Joseph et al., (2021) |
| Positive cognition |  |  | Psychological Performance Inventory - Alternative | Joseph et al., (2021) |
| Visualisation |  |  | Psychological Performance Inventory - Alternative | Joseph et al., (2021) |
| Challenge |  |  | Mental Toughness Questionnaire-48 | Tredrea et al., (2017) |
| Commitment |  |  | Mental Toughness Questionnaire-48 | Tredrea et al., (2017) |
| Control (life) |  |  | Mental Toughness Questionnaire-48 | Tredrea et al., (2017) |
| Grit |  |  | Short Grit Scale | Larkin et al., (2023) |
| Consistency of effort |  |  | Short Grit Scale | Larkin et al., (2023) |
| Perseverance of effort |  |  | Short Grit Scale | Larkin et al., (2023) |
| Emotional stress |  | **b1390 Global mental functions, others specified, psychological wellbeing** | Recovery-stress questionnaire for athletes | Ivarsson et al., (2015) |
| General stress |  |  |  | Rogen et al., (2020) |
| Sport stress |  |  |  |  |
| General recovery |  |  |  |  |
| Sport recovery |  |  |  |  |
| Psychological wellbeing |  |  | General Health Questionnaire-12 | Ivarsson et al., (2015) |
|  |  |  | KIDSCREEN-27 | Rogen et al., (2020) |
| Burnout  (inc. exhaustion)  (psychophysiological) |  | **b1390 Global mental functions, others specified, burnout** | Athlete Burnout Questionnaire | Adie et al., (2012)  Cheval et al., (2017)  Curran et al., (2013) |
| Concentration | **b140 Attention functions** | **b1400 Sustaining attention** | Athletic Coping Skills Inventory-28 | Csáki et al., (2017) |
|  |  |  | Psychological Skills Inventory for Sports | Elferink-Gemser et al., (2004)  Elferink-Gemser et al., (2007)  Huijgen et al., (2014) |
|  |  |  | 5C questionnaire | Harwood et al., (2015) |
|  |  |  | Combined observations and questionnaires | Bolckmans et al. (2023) |
| Psychological distress | **b152 Emotional functions** | **b1520 Appropriateness of** **emotion** | General Health Questionnaire-12 | Blakelock et al., (2016) |
| Anxiety control |  | **b1521 Regulation of emotion** | Psychological Skills Inventory for Sports | Elferink-Gemser et al., (2004)  Elferink-Gemser et al., (2007)  Huijgen et al., (2014) |
| Cognitive anxiety |  |  | Competitive State Anxiety Inventory-2 | Reilly et al., (2000) |
| Somatic anxiety intensity |  |  | Competitive State Anxiety Inventory-2 | Reilly et al., (2000) |
| Coping with performance and developmental pressures |  |  | Psychological Characteristics for developing excellence | Saward et al., (2019)  Kelly et al., (2020)  Kelly et al., (2023)  Hauw et al., (2022) |
| Adverse response to failure |  |  | Psychological Characteristics of Developing Excellence Questionnaire-version 2 | Hill et al., (2019)  Barraclough et al. (2022) |
| Active coping |  |  | Psychological Characteristics of Developing Excellence Questionnaire-version 2 | Hill et al., (2019)  Barraclough et al. (2022) |
| Control (emotions) |  |  | Mental Toughness Questionnaire-48 | Tredrea et al., (2017) |
| Managing emotion |  |  | Combined observations and questionnaires | Bolckmans et al. (2023) |
| Coping with adversity |  |  | Athletic Coping Skills Inventory Coping Skills-28 | Csáki et al., (2017) |
| Peaking under pressure |  |  |  |  |
| Freedom from worry |  |  |  |  |
| Disruption of concentration |  |  | Competition Anxiety Inventory- Trait | Wachsmuth et al., (2023) |
| Self-confidence intensity |  | **b1522 Range of emotion** | Competitive State Anxiety Inventory-2 | Reilly et al., (2000) |
| Cognitive anxiety direction |  |  |  |  |
| Somatic anxiety direction |  |  |  |  |
| Self-confidence direction |  |  |  |  |
| Somatic anxiety |  |  | Competition Anxiety Inventory- Trait | Wachsmuth et al., (2023) |
| Tension |  |  | Profile of Mood States | Laurin et al., (2008) |
| Depression |  |  |  |  |
| Hostility |  |  |  |  |
| Vigor |  |  |  |  |
| Fatigue |  |  |  |  |
| Confusion |  |  |  |  |
| Anxiety |  |  | Global Anxiety Disorder 7-item scale | Hauw et al., (2022) |
|  |  |  | General Health Questionnaire-12 | Ivarsson et al., (2015)  Jordana et al., (2022) |
|  |  |  | Sport Emotion Questionnaire | Turner et al., (2013) |
| Excitement |  |  |  |  |
| Happiness |  |  |  |  |
| Negative affect |  |  | Affective responses tool | Rumbold et al., (2020) |
| Positive affect |  |  | Affective responses tool | Rumbold et al., (2020) |
| Worry |  |  | Competition Anxiety Inventory- Trait | Wachsmuth et al., (2023) |
| Self-control | **b160 Thought functions** | **b1603 Control of thought** | Brief self-control scale | Wolff et al., (2019) |
| Control |  |  | 5C questionnaire | Harwood et al., (2015) |
|  |  |  | Academic Control Scale | Turner et al., (2013) |
| Self-optimisation |  |  | Volitional Components in Sport | Feichtinger & Honer (2015)  Wachsmuth et al., (2023) |
| Self-impediment |  |  |  |  |
| Loss of focus |  |  |  |  |
| Threat appraisals | **b163 Basic cognitive functions** | **b1630 Basic cognitive functions, appraisals** | Cognitive appraisal tool | Rumbold et al., (2020) |
| Challenge appraisals |  |  |  |  |
| Harm appraisals |  |  |  |  |
| Planning | **b164 Higher-level cognitive functions** | **b1641 Organization and planning** | Football-Specific Self-Regulated Learning Questionnaire | Cumming et al., (2018) |
|  |  |  | Self-Regulation of Learning Self-Report Scale | Toering et al., (2012) |
| Goal setting |  |  | Athletic Coping Skills Inventory Coping Skills-28 | Csáki et al., (2017) |
| Mental preparation |  |  | Psychological Skills Inventory for Sports | Elferink-Gemser et al., (2004)  Elferink-Gemser et al., (2007)  Huijgen et al., (2014) |
| Imagery use during practice and competition |  |  | Psychological Characteristics for developing excellence | Saward et al., (2019)  Kelly et al., (2020)  Kelly et al., (2023)  Hauw et al., (2022) |
| Imagery and active preparation |  |  | Psychological Characteristics of Developing Excellence Questionnaire-version 2 | Hill et al., (2019)  Barraclough et al. (2022) |
| Self-monitoring |  | **b1644 Insight** | Self-Regulation of Learning Self-Report Scale | Toering et al., (2012) |
| Evaluation |  | **b1645 Judgement** | Football-Specific Self-Regulated Learning Questionnaire | Cumming et al., (2018) |
|  |  |  | Self-Regulation of Learning Self-Report Scale | Toering et al., (2012) |
| Reflection |  |  | Football-Specific Self-Regulated Learning Questionnaire | Cumming et al., (2018) |
|  |  |  | Self-Regulation of Learning Self-Report Scale | Toering et al., (2012) |
| Executing control to express affection |  | **b1643 Cognitive flexibility** | Coping functions and behaviours tool | Rumbold et al., (2020) |
| Executing control to solve problems |  | **b1646 Problem-solving** | Coping functions and behaviours tool | Rumbold et al., (2020) |
| Eliciting support to solve problems |  |  |  |  |
| Self-regulation learning (Global Score) |  | **b1648 Higher-level cognitive functions, other specified, self-regulation** | Football-Specific Self-Regulated Learning Questionnaire | Cumming et al., (2018)  Erikstad et al., (2018) |
|  |  |  | Self-Regulation of Learning Self-Report Scale | Toering et al., (2012) |
| Evaluating performances and working on weaknesses |  |  | Psychological Characteristics for developing excellence | Saward et al., (2019)  Kelly et al., (2020)  Kelly et al., (2023)  Hauw et al., (2022) |
| Self-directed control and management |  |  | Psychological Characteristics of Developing Excellence Questionnaire-version 2 | Hill et al., (2019)  Barraclough et al. (2022) |
| Self-development |  |  | Combined observations and questionnaires | Bolckmans et al. (2023) |
